# Dietary supplementation of 3′-sialyllactose or 6′-sialyllactose elicits minimal influence on cognitive and brain development in growing pigs

**DOI:** 10.3389/fnbeh.2023.1337897

**Published:** 2024-01-10

**Authors:** Rebecca K. Golden, Loretta T. Sutkus, Sharon M. Donovan, Ryan N. Dilger

**Affiliations:** ^1^Neuroscience Program, University of Illinois, Urbana, IL, United States; ^2^Department of Food Science and Human Nutrition, University of Illinois, Urbana, IL, United States; ^3^Division of Nutritional Sciences, University of Illinois, Urbana, IL, United States; ^4^Department of Animal Sciences, University of Illinois, Urbana, IL, United States

**Keywords:** cognition, brain development, MRI, sialic acid, 3′-sialyllactose, 6′-sialyllactose

## Abstract

Sialylated human milk oligosaccharides (HMO), such as 3′-sialyllactose (3′-SL) and 6′-sialyllactose (6′-SL), are abundant throughout lactation and at much higher concentrations than are present in bovine milk or infant formulas. Previous studies have suggested that sialylated HMO may have neurocognitive benefits in early life. Recent research has focused on infant formula supplementation with key nutrients and bioactives to narrow the developmental gap between formula-fed and breastfed infants. Herein, we investigated the impact of supplemental 3′-SL or 6′-SL on cognitive and brain development at two time-points [postnatal days (PND) 33 and 61]. Two-day-old piglets (*N* = 75) were randomly assigned to commercial milk replacer *ad libitum* without or with 3′-SL or 6′-SL (added in a powdered form at a rate of 0.2673% on an as-is weight basis). Cognitive development was assessed via novel object recognition and results were not significant at both time-points (*p* > 0.05). Magnetic resonance imaging was used to assess structural brain development. Results varied between scan type, diet, and time-point. A main effect of diet was observed for absolute volume of white matter and 9 other regions of interest (ROI), as well as for relative volume of the pons on PND 30 (*p* < 0.05). Similar effects were observed on PND 58. Diffusion tensor imaging indicated minimal differences on PND 30 (*p* > 0.05). However, several dietary differences across the diffusion outcomes were observed on PND 58 (*p* < 0.05) indicating dietary impacts on brain microstructure. Minimal dietary differences were observed from myelin water fraction imaging at either time-point. Overall, sialyllactose supplementation had no effects on learning and memory as assessed by novel object recognition, but may influence temporally-dependent aspects of brain development.

## 1 Introduction

Research has proven that human milk is superior to infant formula in the first year of life, supporting improved short- and long-term health outcomes. Specifically, discrepancies have been found in cognitive and brain development between breastfed and formula-fed infants (Lönnerdal, 2016; Martin et al., 2016). These differences are largely attributed to the compositional differences between human milk and formula. While human milk composition varies by stage of lactation, maternal diet, and genetics, formula composition is static and designed to meet nutrient requirements of the infant, and can be devoid of bioactive components (Michaelsen et al., 1990; Koletzko et al., 2005; Fujita et al., 2012). A key component of high interest is the human milk oligosaccharide (HMO) fraction, which is composed of monosaccharides such as *N*-acetylglucosamine (Neu5Gc) and *N*-acetylneuraminic acid (Neu5Ac), both of which are sialic acids (SA) (Bode, 2012; Andreas et al., 2015). HMO play a vital role in infant development supporting growth of beneficial bacteria, acting as prebiotic substances, preventing infection, and supporting brain and cognitive development (Ten Bruggencate et al., 2014; Hobbs et al., 2021). Despite the reported benefits of HMO, infant products are typically formulated using bovine milk, containing a very small quantity of oligosaccharides that differ in composition and concentration compared with human milk. Whereas human milk typically contains a total oligosaccharide concentration of 3.5–14 g/L, bovine milk contains just 0.3–0.5 g/L. This discrepancy leads to a large oligosaccharide concentration range for infant formulas, spanning anywhere from 0.4 to 8 g/L (Coppa et al., 2004; Ten Bruggencate et al., 2014; Hobbs et al., 2021).

Elucidating the longitudinal influence of HMO, consumed individually or in mixtures, is of increasing importance due to the high concentrations of endogenous HMO-related compounds found in the brain throughout the lifespan. Recent research has been focused on the impact of ingesting sialylated oligosaccharides [i.e., sialyllactose (SL)], the most prominent forms of which are 3′-sialyllactose (3′-SL) and 6′-sialyllactose (6′-SL) (Tarr et al., 2015). Of the SL found in HMO, the most prevalent backbone is Neu5Ac, which is thought to play a role in supporting neural communication, synaptogenesis, brain growth, and cognitive ability (Wang et al., 2001; Wang and Brand-Miller, 2003). Importantly, the composition of human milk changes throughout lactation in accordance with the nutritional needs of the infant. Of note, there are distinct differences in the prevalence of 3′-SL and 6′-SL throughout lactation, with 3′-SL increasing and 6′-SL decreasing in concentration during this period (Plows et al., 2021). Some rodent studies have supported the positive impact of Neu5Ac supplementation, noting increases in learning abilities and uptake of gangliosides in the brain (Morgan and Winick, 1980), while others have shown null effects, observing no cognitive benefit from Neu5Ac supplementation (Oliveros et al., 2018). Similarly, reported findings across different species disagree on the impact of SL supplementation on cognition with some studies indicating null or ambiguous effects (Sakai et al., 2006; Fleming et al., 2018; Oliveros et al., 2018) and others indicating positive effects (Obelitz-Ryom et al., 2019; Docq et al., 2020; Cho et al., 2021; Hauser et al., 2021). The impact of SL supplementation on brain development is equally as complicated. While SL supplementation does not seem to influence absolute brain weight, brain volume, or cell size/number (Morgan and Winick, 1980; Sakai et al., 2006; Mudd et al., 2017), a closer examination indicates scan-dependent, region-specific brain development differences. Specifically, differences have been observed in the hippocampus, corpus callosum, prefrontal cortex, and total white matter volume (Mudd et al., 2017; Obelitz-Ryom et al., 2019; Wang et al., 2021) indicating potential sensitivity to dietary SL supplementation.

As such, the objective of this study was to evaluate the impact of pure 3′-SL or 6′-SL supplementation on cognitive and brain development in pigs, both in the short- and long-term. We hypothesized that both 3′-SL and 6′-SL supplemented pigs would perform better in an object recognition task, indicating more advanced cognitive development. We also predicted that while overall brain volumes would not differ, region-specific differences would emerge with the supplemented pigs having more myelination in white-matter dense regions.

## 2 Materials and methods

### 2.1 Animals, housing, and diet groups

A total of seventy-five intact (i.e., non-castrated) male pigs were obtained from a commercial swine farm on postnatal day (PND) 2 and transported to the University of Illinois Piglet Nutrition and Cognition Lab (PNCL) in cohorts of 15. The utilized number of pigs provided 80% power to detect 1.25 standard deviation units with a 5% level of significance (i.e., alpha = 0.05) between treatment groups as determined by a power analysis via SAS (RRID:SCR_008567; version 9.3; SAS Inst. Inc., Cary, NC, United States). Genetic variability was minimized via Pig Improvement Company (PIC; Hendersonville, TN) Line 3 dams that were artificially inseminated using a pooled semen source from 50 to 150 boars. Upon arrival at PNCL, pigs received a 3-mL subcutaneous and 3-mL oral dose of *Clostridium perfringens* antitoxin C and D (Colorado Serum Company, Denver, CO). Pigs were individually, artificially reared at PNCL until PND 33, at which point 6 pigs (2 from each diet) were randomly selected for baseline sample collection. The remaining 9 pigs were transported to the Veterinary Medicine Research Farm (VMRF) and group-raised in pens (*n* = 3 per treatment in each pen) until study conclusion on PND 61.

Individual pig-rearing units and housing conditions at PNCL have been previously described in detail (Fil et al., 2021b). The units are designed to allow pigs to see, hear, and smell each other, but prohibit direct touching. Health checks, feed disappearance, and body weights were captured daily. Pigs had access to electrolytes (Swine BlueLite; TechMix, Stewart, MN or Bounce Back, MannaPro®, LLC, St. Louis, MO) for the first week at PNCL, regardless of diet, to maintain hydration as piglets adapted to the formulas. Outcomes associated with growth performance, fecal consistency, and health status have been described separately (Golden et al., 2023).

#### 2.1.1 Experimental diets

Pigs were randomly assigned to one of three diet groups: a commercial standard milk replacer diet (CON; TestDiet, Purina Mills, St. Louis, MO), CON +3′-sialyllactose (3′-SL), or CON +6′-sialyllactose (6′-SL). From PND 2-33, all diets were based on a commercial milk replacer product (ProNurse^®^ Specialty Milk Replacer, Land O’Lakes, North Arden Hills, MN, United States) and supplemented accordingly. From PND 33-61, diets were manufactured as nutritionally adequate and age-appropriate mash diets (Golden et al., 2023). Pure sialyllactose was added at a rate of 0.2673% of the diet on an as-is basis (PND 2-33: 500 mg/L; PND 33-61: 2.673 g/kg). Although 6′-SL is typically more abundant than 3′-SL in human milk, the concentration of either SL varies greatly between different individuals as well as quantification methods. As such, for the purposes of this study, dosage was determined by averaging the concentrations of both SL across lactation (Soyyilmaz et al., 2021). Dietary composition and the feeding paradigm utilized is described in detail separately (Golden et al., 2023). All listed experimental procedures were approved by the University of Illinois Urbana-Champaign institutional Animal Care and Use Committee and were aligned with the National Research Council Guide for the Care and Use of Laboratory Animals.

### 2.2 Behavioral testing

Behavioral testing was done via the novel object recognition (NOR) task, described in detail previously (Fleming and Dilger, 2017), which relies on the inherent preference pigs have for novelty in their environment. All pigs were subject to behavioral testing from PND 24-28. Longitudinal pigs were tested again from PND 52-56. Each day of the testing process at both time-points was recorded via the Motif video recording system (Loopbio, GmbH, Vienna, Austria). Testing consisted of a habituation phase, a sample phase, and a test phase. During the habituation phase, each pig was placed in an empty arena (72 in × 72 in × 45.5 in; L × W × H; ShapeMaster, Ogden, IL, United States) for 10 min on two consecutive days leading up to the sample phase. In the sample phase, two identical objects were secured to the floor of the arena (center-left and center-right). Each pig was allowed to explore the arena with the objects in it for 5 min. After a 48-h delay, each pig was reintroduced into the arena, now containing one familiar object from the sample phase and one novel object. Pigs were once again allowed to explore the objects and arena for 5 min. Between each pig, the objects and arena were cleaned with diluted bleach and warm water to mitigate odor and remove any excrement. Different sets of objects were used for testing at PND 24 and PND 52 to eliminate any chance of familiarity bias. Which object was designated the sample object or novel object was counterbalanced within each cohort. Similarly, side of the arena the novel object was presented on was also counterbalanced within each cohort. Outcome measures were analyzed by a single trained, blinded study personnel via the use of Loopy, an online video analysis software (http://loopbio.com/recording/, Loopbio GmbH, Vienna, Austria). The main cognitive outcome was recognition index, or the amount of time spent investigating the novel object as a proportion of total investigation time of both objects. Other exploratory measures such as latency to interactions and frequency and duration of interactions were also quantified individually for each object as well as combined for total exploratory behavior. An inclusion criterion of exploration of strictly the novel object for ≥2 s + exploration of strictly the familiar object for ≥2 s was applied before running statistical analyses to reduce bias associated with the incidence of object preference.

### 2.3 Magnetic resonance imaging (MRI)

**Figure 1:**
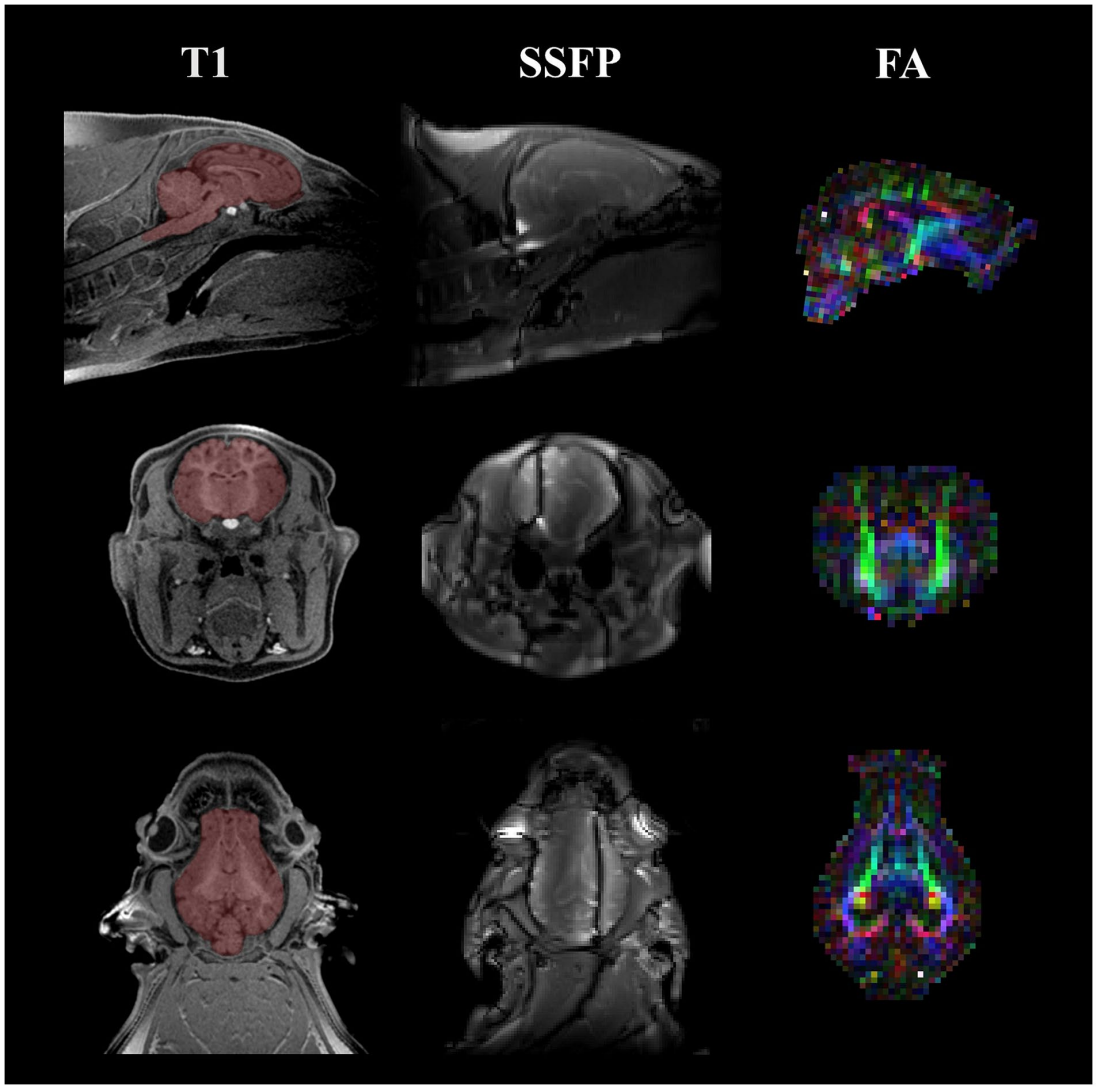
Representative MRI images acquired on 4 wk and 8 wk pigs. Representative images are displayed for the three MRI techniques utilized at both the 4 wk and 8 wk time points. Starting from the left column, a T1-weighted MPRAGE image of a singular pig at the 4-week time point is displayed with a manually traced brain mask overlaid on top of the anatomical image in red. Next, a representative image of one of the SSFP images acquired for calculating myelin water fraction is displayed. Field inhomogeneities are present in the coronal and transverse views. Lastly, the right column showcases a colored FA map acquired from DTI. The different colors in the colored FA map highlight different fiber directions (red, left–right; green, anterior–posterior; blue, superior–inferior). All images include a sagittal, coronal, and transverse view starting from the top of the figure moving down, respectively. MRI, magnetic resonance imaging; MPRAGE, magnetization prepared rapid–gradient echo; SSFP, steady-state free precession; FA, fractional anisotropy; DTI, diffusion tensor imaging.

The 33 (*n* = 11 per treatment) pigs randomly selected to continue to VMRF underwent neuroimaging procedures at the Beckman Institute Biomedical Center using a Siemens MAGNETOM Prisma 3 T MRI. Selected pigs were subjected to scans at two time-points: PND 30 and PND 58. The pig neuroimaging protocol for both time-points included a magnetization prepared rapid gradient-echo (MPRAGE) sequence, diffusion tensor imaging (DTI), and a multicomponent driven equilibrium single pulse observation of T_1_ and T_2_ (mcDESPOT) to assess brain macrostructure, microstructure, and myelin-associated water fraction (MWF), respectively. Representative images of each brain imaging technique are displayed in Figure 1. During scanning procedures, pigs were sedated utilizing an intramuscular injection of a telazol: ketamine: xylazine cocktail [50.0 mg tiletamine plus 50.0 mg of zolazepam reconstituted with 2.50 mL ketamine (100 g/L) and 2.50 mL xylazine (100 g/L); Fort Dodge Animal Health, Overland Park, KS] at 0.03 mL/kg of body weight. Once immobilized, pigs were placed in a supine position with the head placed into a custom-made 8-channel piglet head coil at PND 30 or an 18-channel flex body coil in combination with a 32-channel spine coil for pigs at PND 58. For anesthesia maintenance, inhalation of 2% isoflurane was administered in conjunction with 98% oxygen through a mask tightly fitted on the pig’s snout. Heart rate and oxygen levels were monitored and recorded every 5 min utilizing one to two infrared sensor pulse oximeters (LifeWindow LW9x, Boynton Beach, FL and MEDRAD Veris 8,600, Indianola, PA) clipped onto the pig’s tail and/or hind hoof. The total scan time for each pig was approximately 35 min.

#### 2.3.1 Structural MRI acquisition and analysis

To obtain anatomic images of the pig brain, a T1-weighted MPRAGE sequence was obtained from the tip of the snout to the cervical/thoracic spinal cord junction. At both time points sagittal-oriented data were acquired with a 173 mm × 173 mm × 153.6 mm field of view (FOV) and a 288 × 288 × 256 matrix. The following parameters were used for pigs at both time points: repetition time (TR) = 2,060 ms, echo time (TE) for PND30/PND58 = 2.05 ms/2.52 ms, inversion time (TI) = 1,060 ms, and flip angle (α) = 9° providing a final voxel volume of 0.6 × 0.6 × 0.6 mm^3^. For PND 58, two MPRAGE sequences were acquired, and the images were averaged to obtain better signal to noise ratio (SNR).

Image processing began with hand-tracing and subsequently extracting each pig brain using FMRIB Software Library (FSL). Once extracted, each pig brain was manually reoriented utilizing Statistical Parametric Mapping version 12 (SPM12; University College London, London, UK) imaging software. The pig brains were further oriented to align with the Pig Brain Atlas version 2.0 (Fil et al., 2021c), utilizing the “Coregister: Estimate” function in SPM12. The 4-week Pig Brain Atlas and 4-week [regions of interest] ROI masks were utilized for PND 30 scans, while the 12-week Pig Brain Atlas and 12-week ROI masks were used for PND 58 scans. Nonlinear registration was performed using “fnirt” and inverse warp options in FSL to manipulate the Pig Brain Atlas and 26 ROI into subject space. Absolute volumes were then calculated for the 26 ROI utilizing “fslmaths” commands and a threshold of 0.5 in FSL. Additionally, the “Segment” function was used in SPM12 with age-dependent, pig-specific, tissue-probability maps to obtain values for gray matter, white matter, and cerebral spinal fluid. To calculate relative volumes for ROI and segmentations, the following equation was used: [(ROI absolute volume) ÷ (whole brain absolute volume)] × 100.

#### 2.3.2 Diffusion tensor imaging acquisition and analysis

To assess white matter maturation and axonal tract integrity, a diffusion-weighted echo planar imaging (DWI-EPI) sequence was utilized (Feinberg et al., 2010; Moeller et al., 2010; Xu et al., 2013). At both time points, transversal-oriented images were acquired with a 160 mm × 160 mm × 80 mm FOV and 100 × 100 × 50 matrix providing a final voxel volume of 1.6 × 1.6 × 1.6 mm^3^. PND 30 and PND 58 scans differed in TR/TE, where at PND 30, TR/TE = 6,180 ms/78.20 ms and at PND 58, TR/TE = 5,100 ms/70 ms. The following parameters were used for both time points: α = 90°, GRAPPA accelerated by a factor of 2 in the phase encode direction, multiband factor of 1, diffusion weightings = 1,000 and 2,000 s/mm^2^ across 30 directions and 1 image with a b-value of 0 s/mm^2^.

After acquisition, image processing began with hand-tracing the images for brain isolation. As previously described, the FSL diffusion toolbox^1^ was utilized to generate values for fractional anisotropy (FA), mean diffusivity (MD), axial diffusivity (AD), and radial diffusivity (RD). Linear and nonlinear transformations were conducted following previously established methods (Fil et al., 2021c). Diffusion values were calculated for overall white matter as well as the following selected white matter ROI: cerebellum, corpus callosum, right/left internal capsules, right/left hippocampi, right/left caudate, and thalamus.

#### 2.3.3 Myelin water fraction acquisition and analysis

To investigate myelin content within the brain, myelin water imaging was conducted. An established method for investigating myelin content throughout the brain is through multicomponent driven equilibrium single pulse observation of T_1_ and T_2_ (mcDESPOT) (Deoni et al., 2008). This technique derives information from multiple sets of spoiled gradient-recalled echo (SPGR) and T_2_/T_1_-weighted balanced steady-state free precession (SSFP) data that are acquired at varying flip angles (α) and with a constant repetition time (TR). With this data, T_2_, T_1_, water volume fractions and water residence time estimates can be obtained of the extracellular and intracellular pools of water that are within the lipid bilayers of myelin.

At both time points, sagittal-oriented data were acquired with a 160 mm × 160 mm × 124.8 mm FOV and a 128 × 128 × 96 matrix. To accommodate main magnetic field inhomogeneities, SSFP data was acquired with two phase-cycling increments of 0° and 180° data with the following parameters: TR/TE = 5.3 ms/2.65 ms, α = (11, 15, 19, 23, 27, 35, 50, and 70)°, and bandwidth (BW) = 350 Hz/Px, providing a final voxel volume of 1.25 × 1.25 × 1.3 mm^3^. Furthermore, SPGR data were acquired with the following parameters: TR/TE = 5.6 ms/2.7 ms, flip angles of (3, 4, 5, 6, 7, 9, 13, and 18)°, and BW = 350 Hz/Px, providing a final voxel volume of 1.25 × 1.25 × 1.3 mm^3^. Additionally, two high resolution T1-weighted inversion recovery (IR)-SPGR sequences were acquired to correct for transmit (B_1_) magnetic field inhomogeneities with two different inversion times. The IR-SPGR parameters were as follows: FOV = 160 mm x 160 mm x 124.8 mm, matrix = 96 × 96 × 48, TR/TE/TI = 5.6 ms/2.7 ms/(450, 750) ms, α = 5°, and BW = 350 Hz/Px, providing a final voxel volume of 1.7 × 1.7 × 2.6 mm^3^.

The MWF images were linearly registered to subject MPRAGE images to ensure proper alignment. Linear and nonlinear transformations were also performed between the atlas and MPRAGE images to register images into the same space. Using the warp outputs and the coordinates obtained, the Pig Brain Atlas was successfully transformed into MWF space. A threshold of 0.5 was applied to all 26 ROI and the resulting total MWF image after running mcDESPOT (Deoni et al., 2008) was used to calculate mean MWF. The 4-week Pig Brain Atlas and threshold 0.5 ROI masks were used for PND 30 scans, while the 12-week Pig Brain Atlas and 12-week threshold 0.5 ROI masks were used for PND 58 scans (Fil et al., 2021c).

### 2.4 Statistical analysis

Behavioral and MRI outcomes were analyzed by a one-way analysis of variance (ANOVA) utilizing the mixed procedure in SAS (RRID:SCR_008567; version 9.3; SAS Inst. Inc., Cary, NC, United States). Four- and eight-week outcomes were analyzed and reported separately for both NOR and MRI due to the changes in testing location for NOR and neuroimaging coil for MRI. Therefore, longitudinal behavioral assessments and changes in MRI outcomes were not captured. For recognition memory, a one-sample *t*-test was utilized, where recognition index was compared with a chance performance value of 0.50. For all other statistical outcomes, the main effect of dietary treatment was assessed using cohort as a blocking factor, with litter of origin nested within cohort, to control both sources of variance. Level of significance was set at *p* < 0.05 for all analyses. Outlier removal was performed for all measures and determined by results producing a studentized residual of ±3.

## 3 Results

### 3.1 Behavioral outcomes

Behavioral outcomes were analyzed by housing location due to minor differences in the testing arena setups. Mainly, the testing environment at PNCL had rounded corners, whereas the testing environment at VMRF did not. Although quantitative proof is not available, anecdotal evidence suggests that the pigs behaved slightly differently in the environments, with pigs expressing more rooting behavior in the unrounded corners of the VMRF arena. Any pig expressing mobility problems or sickness either before or during any part of the task was removed from testing and any data collected was deleted. This lead to the exclusion of 4 pigs at PND 28 and 3 pigs at PND 56. As such, 61 and 33 pigs completed the NOR task at PND 28 and PND 56, respectively. Prior to statistical analysis, an inclusion criterion of interaction of at least 2 s with both the novel object and the sample object was applied to reduce bias associated with object preference. As such, 52 and 31 pigs were included in the final dataset for PND 28 and PND 56, respectively. Along with recognition index (RI), exploratory measures were also quantified, including average object visit time, total object visit time, number of object visits, latency to first object visit, and latency to last object visit. Exploratory measures were divided into novel object exploration, sample object exploration, and both objects exploration.

**Figure 2:**
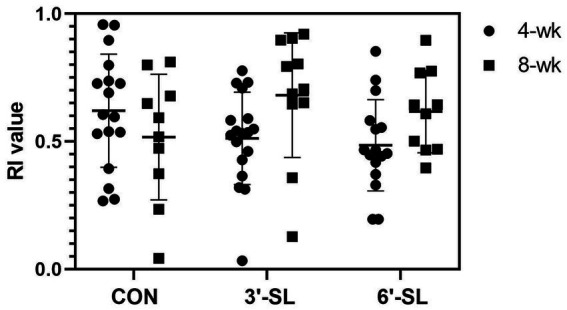
Variability in RI values by treatment and testing week. Variability of RI values produced at both the 4-wk and 8-wk time-point are displayed by treatment. RI, recognition index; CON, dietary group given a control diet; 3′-SL, dietary group supplemented with 0.273% 3′-S; 6′-SL, dietary group supplemented with 0.273% 6′-SL.

At PND 28, a main effect of diet (*p* = 0.016) was observed for latency to the last interaction with either object, with 3′-SL pigs interacting later than the CON pigs and 6′-SL pigs exhibiting intermediary latency to last object interaction (Table 1). While there was no statistical difference between RI values between dietary treatments, the RI value for CON pigs differed from that of the chance value (0.50). The value 0.50 for chance was dictated by the presence of two objects available for exploration. Although neither differed from that of chance, 3′-SL pigs expressed an RI value numerically above 0.50 while 6′-SL pigs exhibited an RI value that was numerically lower than 0.50. There were no main effects of diet observed for any measures at PND 56 (Table 2). In this instance, while RI values for all treatments were above the chance level, only the RI values for 3′-SL and 6′-SL pigs differed (*p* < 0.05). Variability among RI values for all treatments and both time-points are displayed in Figure 2.

**Table 1:** Exploratory behavior of pigs on PND 28 during the test trial of the novel object recognition (NOR) task.^1^

	Diet			Pooled SEM	
Behavioral measure	CON	3′-SL	6′-SL		*p*-value^2^
*n*	17	19	16	–	–
Recognition index	0.63^†^	0.52	0.46	0.06	0.099
Exploration of the *novel* object					
Object visit time, s	35.27	38.36	26.76	7.14	0.481
Number of object visits	7.6	7.8	6.1	0.92	0.254
Mean object visit time, s	4.86	5.66	3.42	0.94	0.233
Latency to first object visit, s	19.07	26.22	31.5	7.13	0.445
Latency to last object visit, s	192.89	239.35	222.05	16.83	0.147
Exploration of the *sample* object					
Object visit time, s	16.07	29.75	27.79	4.97	0.063
Number of object visits	4.9	6.9	6.8	0.69	0.078
Mean object visit time, s	3.13	4.51	4.15	0.62	0.178
Latency to first object visit, s	54.58	23.03	23.27	11.67	0.051
Latency to last object visit, s	210.45	252.67	235.39	16.6	0.197
Exploration of *both* objects					
Object visit time, s	51.77	74.49	54.63	10.2	0.238
Number of object visits	12.4	14.6	12.7	1.24	0.315
Mean object visit time, s	4.15	5.08	4.27	0.71	0.543
Latency to first object visit, s	11.67	9.22	21.12	6.75	0.391
Latency to last object visit, s	240.78^b^	284.05^a^	257.21^ab^	10.04	0.016

^ab^Means lacking a common superscript letter within a row differ (*p* < 0.05).

1 CON, control; 3′-SL, 3′-sialyllactose; 6′-SL, 6′-sialyllactose; SEM, standard error of mean.

2 *p*-value for the overall 1-way ANOVA, which included a single fixed effect of treatment group.

† Recognition index value is different (*p* < 0.05) from that of chance (0.50).

**Table 2:** Exploratory behavior of pigs on PND 56 during the test trial of the novel object recognition (NOR) task.^1^

	Diet			Pooled SEM	
Behavioral measures	CON	3′-SL	6′-SL		*p*-value^2^
*n*	10	11	10	–	–
Recognition index	0.52	0.68^†^	0.62^†^	0.07	0.253
Exploration of the *novel* object					
Object visit time, s	28.43	45.25	37.03	10.32	0.489
Number of object visits	5.5	7.2	7	1.26	0.586
Mean object visit time, s	5.08	5.85	5.18	1.38	0.887
Latency to first object visit, s	31.73	15.16	44.7	11.86	0.214
Latency to last object visit, s	206.06	243.8	26.47	21.96	0.186
Exploration of the *sample* object					
Object visit time, s	22.43	12.12	23.23	5.34	0.257
Number of object visits	6.7	6.4	6.5	1	0.971
Mean object visit time, s	3.49	1.95	2.59	0.47	0.059
Latency to first object visit, s	14.42	27.75	16.86	8.7	0.470
Latency to last object visit, s	213.64	219.34	235.5	23.44	0.827
Exploration of *both* objects					
Object visit time, s	50.48	57.26	59.87	13.11	0.854
Number of object visits	12.2	13.5	13.5	1.9	0.850
Mean object visit time, s	3.39	3.86	4.21	0.85	0.617
Latency to first object visit, s	8.47	8.64	9.64	5.08	0.984
Latency to last object visit, s	242.99	269.4	270.15	14.63	0.308

1 CON, control; 3′-SL, 3′-sialyllactose; 6′-SL, 6′-sialyllactose; SEM, standard error of mean.

2 *p*-value for the overall 1-way ANOVA, which included a single fixed effect of treatment group.

† Recognition index value is different (*p* < 0.05) from that of chance (0.50).

### 3.2 MRI outcomes

A total of 34 pigs successfully underwent MRI procedures at both time-points. Numbers for individual outcomes may vary slightly due to outlier removal and processing issues. Improper scanning parameters on PND 30 led to the removal of 13 pigs from DTI analysis. All sample sizes are listed with their respective outcomes in the corresponding tables.

#### 3.2.1 Absolute and relative volume outcomes

**Figure 3:**
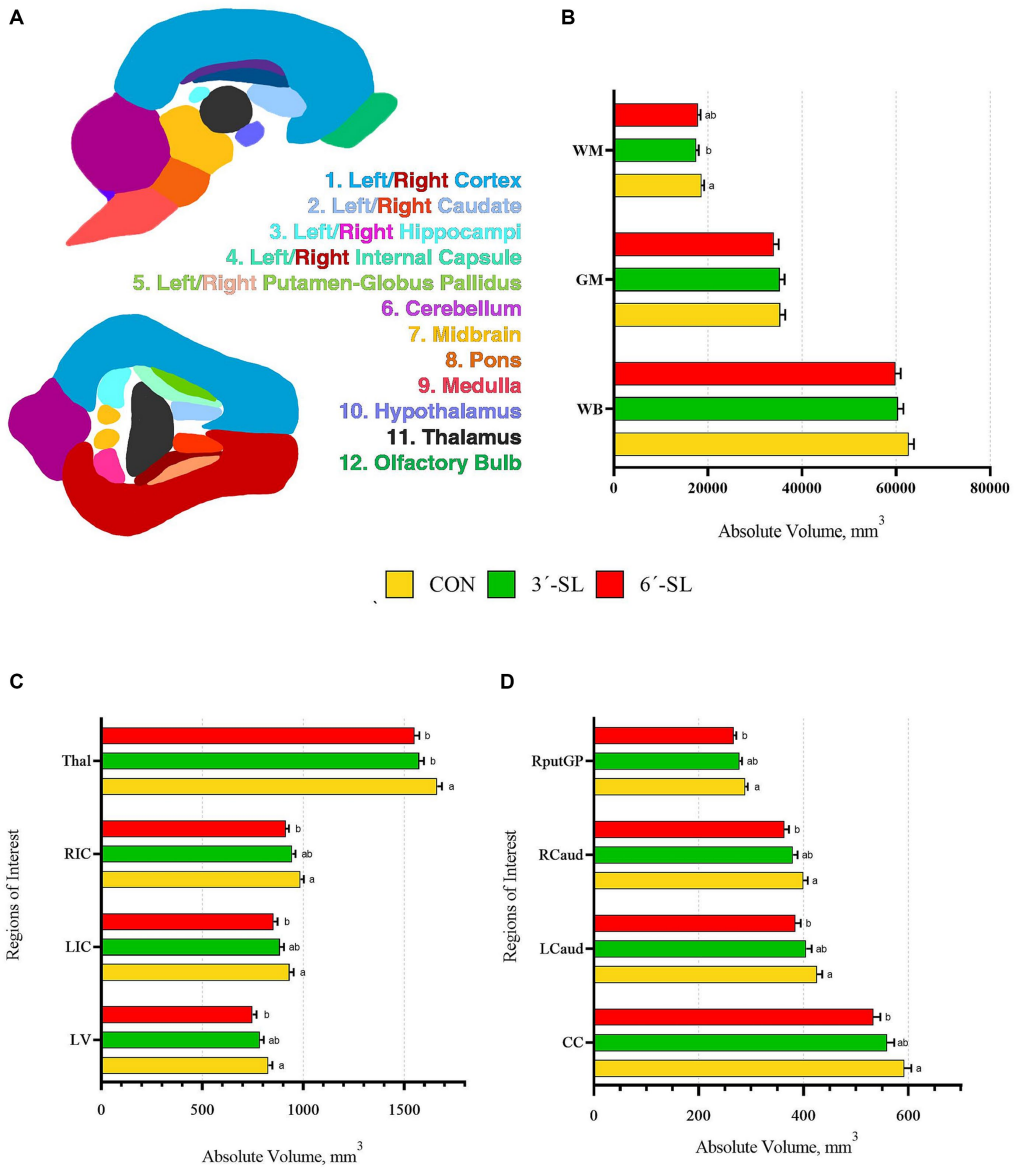
Absolute brain volumes of pigs on PND 30. **(A)** Sagittal and transverse views of the pig brain atlas with color-coded regions of interest are displayed for reference. **(B)** Absolute volumes at PND 30 are displayed for the white matter, gray matter, and whole brain. **(C)** Absolute volumes are displayed for the thalamus (Thal), right internal capsule (RIC), left internal capsule (LIC), and lateral ventricle (LV). **(D)** Absolute volumes are displayed for the right putamen-globus pallidus (RputGP), right caudate (Rcaud), left caudate (Lcaud), and corpus callosum (CC). PND, postnatal day; CON, dietary group given a control diet; 3′-SL, dietary group supplemented with 0.273% 3′-S; 6′-SL, dietary group supplemented with 0.273% 6′-SL. ^ab^Means lacking a common superscript letter differ (*p* < 0.05).

**Figure 4:**
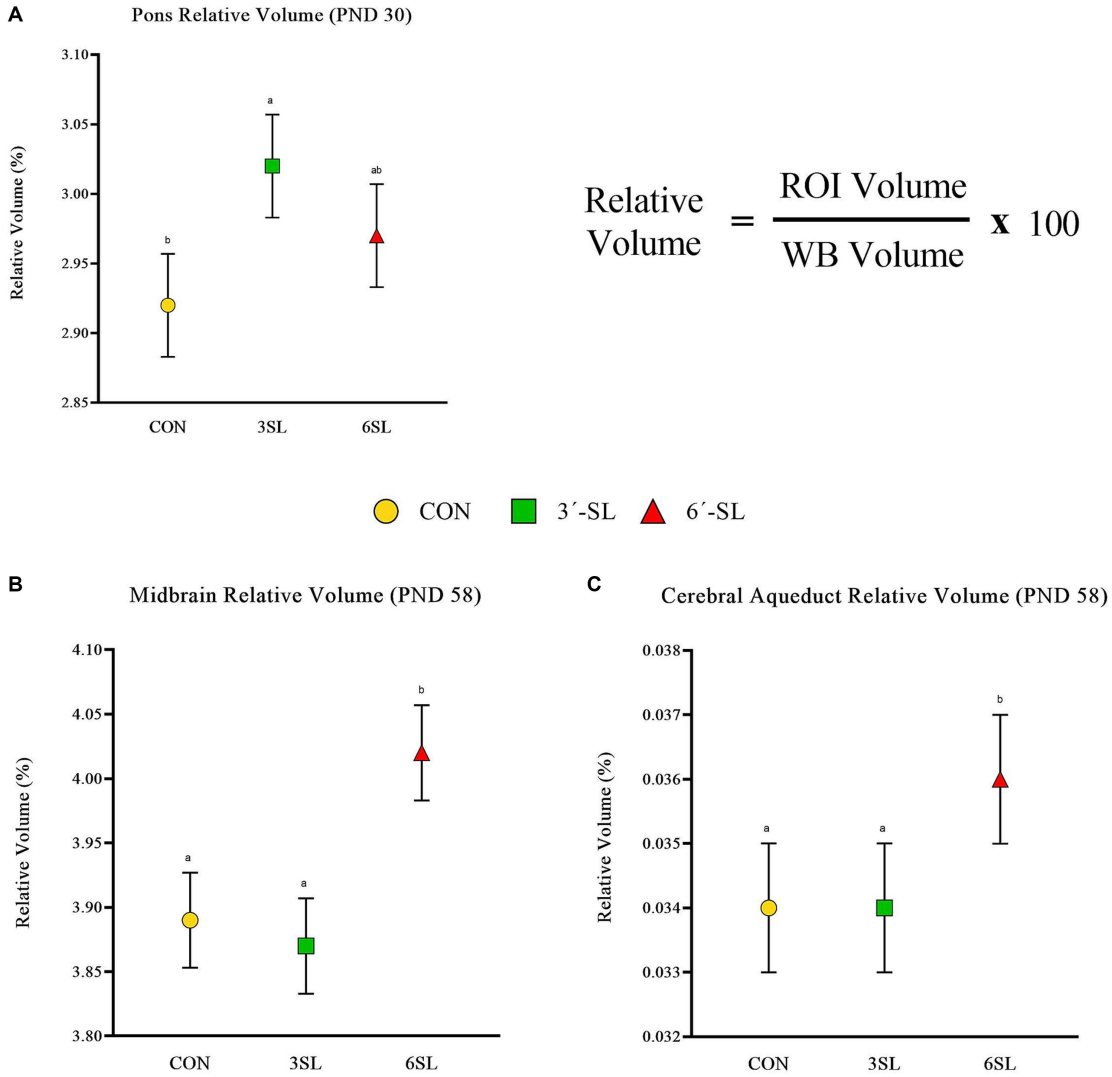
Relative brain volumes of pigs on PND 30 and PND 58. **(A)** Relative volume at PND 30 for the pons brain region is displayed as well as the relative volume equation for reference. **(B)** Relative volume at PND 58 for the midbrain is displayed. **(C)** Relative volume at PND 58 for the cerebral aqueduct is displayed. PND, postnatal day; CON, dietary group given a control diet; 3′-SL, dietary group supplemented with 0.273% 3′-S; 6′-SL, dietary group supplemented with 0.273% 6′-SL; ROI, regions of interest; WB, whole brain. ^ab^Means lacking a common superscript letter differ (*p* < 0.05).

Absolute and relative volumes were obtained for the whole brain, gray matter, white matter, cerebral spinal fluid, and 26 additional regions of interest at both time-points. A main effect of diet was observed across several ROI on PND 30 (Figures 3, 4; Supplementary Tables S1, S2). Differences in absolute white matter volume (*p* = 0.043) were observed, where CON pigs had larger volume on average compared with the 3′-SL group. Additionally, dietary effects were observed for the corpus callosum, lateral ventricle, left and right caudate, left and right internal capsules, and left and right putamen/globus pallidus (all *p* < 0.05). For all listed regions, the CON group was observed to have larger absolute volume compared with the 6′-SL group. A diet effect was also observed for the thalamus (*p* = 0.009), where the CON group had larger absolute volume in this region compared with both the 3′-SL and 6′-SL groups. Differences were observed for the relative volume of the pons brain region (*p* = 0.050), where the 3′-SL group had larger relative volume compared with the CON group. No other relative volume differences between diets were observed across the remaining ROI or segmentations.

**Figure 5:**
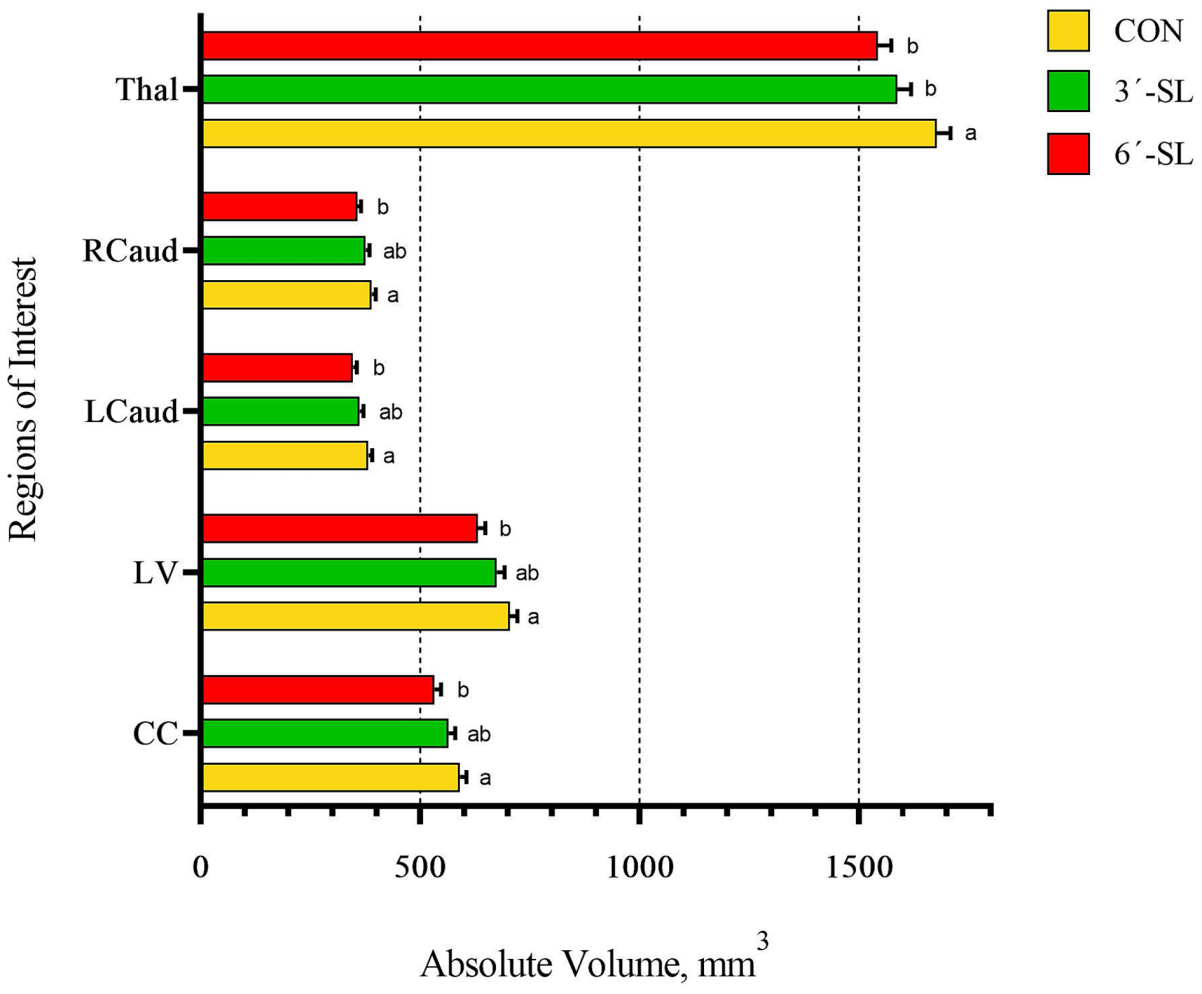
Absolute brain volumes of pigs on PND 58. Absolute volumes at PND 58 for the thalamus (Thal), right caudate (Rcaud), left caudate (Lcaud), lateral ventricle (LV), and corpus callosum (CC) are displayed. PND, postnatal day; CON, dietary group given a control diet; 3′-SL, dietary group supplemented with 0.273% 3′-S; 6′-SL, dietary group supplemented with 0.273% 6′-SL. ^ab^Means lacking a common superscript letter differ (*p* < 0.05).

A main effect of diet was observed for several neuroimaging outcomes at PND 58 (Figures 4, 5; Supplementary Tables S3, S4). Relatively consistent with findings on PND 30, differences (all *p* < 0.05) were observed in the corpus callosum, lateral ventricle, and left and right caudate. Again, for all listed regions, the CON group was observed to have larger absolute volumes compared with the 6′-SL group. Similarly, a diet effect was observed for the thalamus (*p* = 0.018), where the CON group had larger absolute volume compared with the other two dietary treatment groups. A diet effect was observed for relative volume of the cerebral aqueduct (*p* = 0.021) and midbrain (*p* = 0.015), with the 6′-SL group having larger relative volume in both of these regions compared with both the CON and 3′-SL groups.

#### 3.2.2 Diffusion tensor imaging outcomes

**Figure 6:**
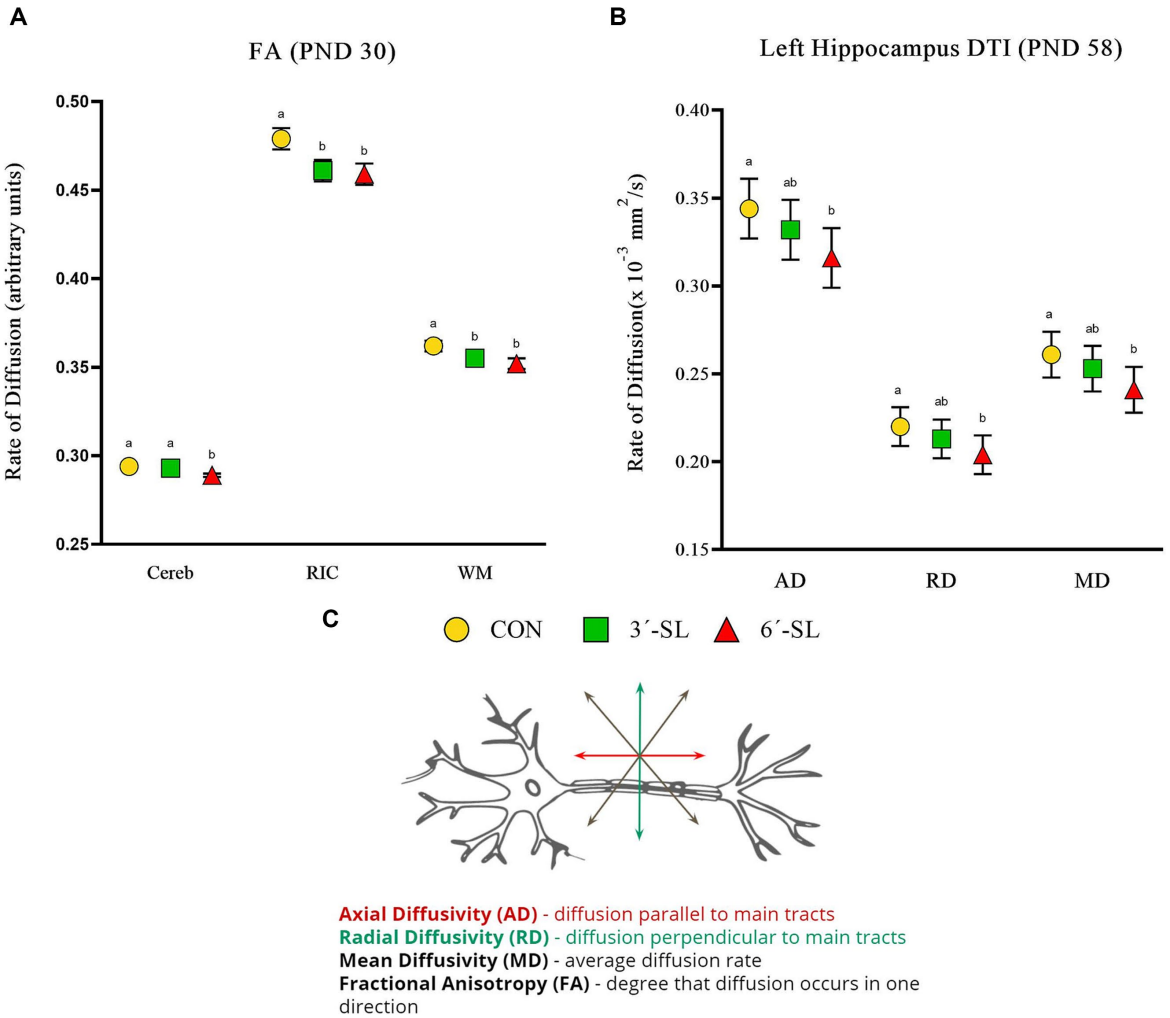
Diffusion tensor imaging (DTI) outcomes in pigs on PND 30 and PND 58. **(A)** Fractional anisotropy values at PND 30 are displayed for the cerebellum (Cereb), right internal capsule (RIC), and overall white matter (WM). **(B)** Diffusivity values for the left hippocampus at PND 58 are displayed. **(C)** A neuron with diffusion directionality is displayed for reference. PND, postnatal day; DTI, diffusion tensor imaging; AD, axial diffusivity; RD, radial diffusivity; MD, mean diffusivity; CON, dietary group given a control diet; 3′-SL, dietary group supplemented with 0.273% 3′-S; 6′-SL, dietary group supplemented with 0.273% 6′-SL; ROI, regions of interest; WB, whole brain. ^ab^Means lacking a common superscript letter differ (*p* < 0.05).

Diffusion tensor imaging values (mm^2^/s) for axial diffusivity (AD), radial diffusivity (RD), mean diffusivity (MD), and fractional anisotropy (FA) were obtained for the overall brain, overall white matter, and 11 distinct regions of interest. Scans on PND 30 showed no diet differences for AD, RD, or MD outcomes (Supplementary Tables S5–S7). Dietary differences (*p* < 0.05) in FA were observed in the cerebellum, right internal capsule, and overall white matter (Figure 6; Supplementary Table S8). The 6′-SL group was observed to have lower FA values (*p* = 0.005) for the cerebellum compared with both the CON and 3′-SL groups. For both the internal capsule (*p* = 0.030) and overall white matter (*p* = 0.047), the CON pigs were observed to have higher FA values than pigs in either the 3′-SL or 6′-SL groups.

Diet differences were observed on PND 58 across all diffusion outcomes (Figure 6; Supplementary Tables S9–S12). For both AD (*p* = 0.032) and MD (*p* = 0.048) in the cerebellum, the 6′-SL group was observed to have lower values compared with either the CON or 3′-SL groups. Diet differences in AD (*p* = 0.010), RD (*p* = 0.026), and MD (*p* = 0.017) were observed for the left hippocampus, where the 6′-SL group had lower values compared with the CON group, but not the 3′-SL group. Dietary differences for FA were observed for both the right hippocampus and the thalamus, where the CON group had higher (*p* = 0.033) values for the right hippocampus compared with either the 3′-SL or 6′-SL groups and higher (*p* = 0.011) values for the thalamus compared with the 6′-SL group.

#### 3.2.3 Myelin water fraction outcomes

**Figure 7:**
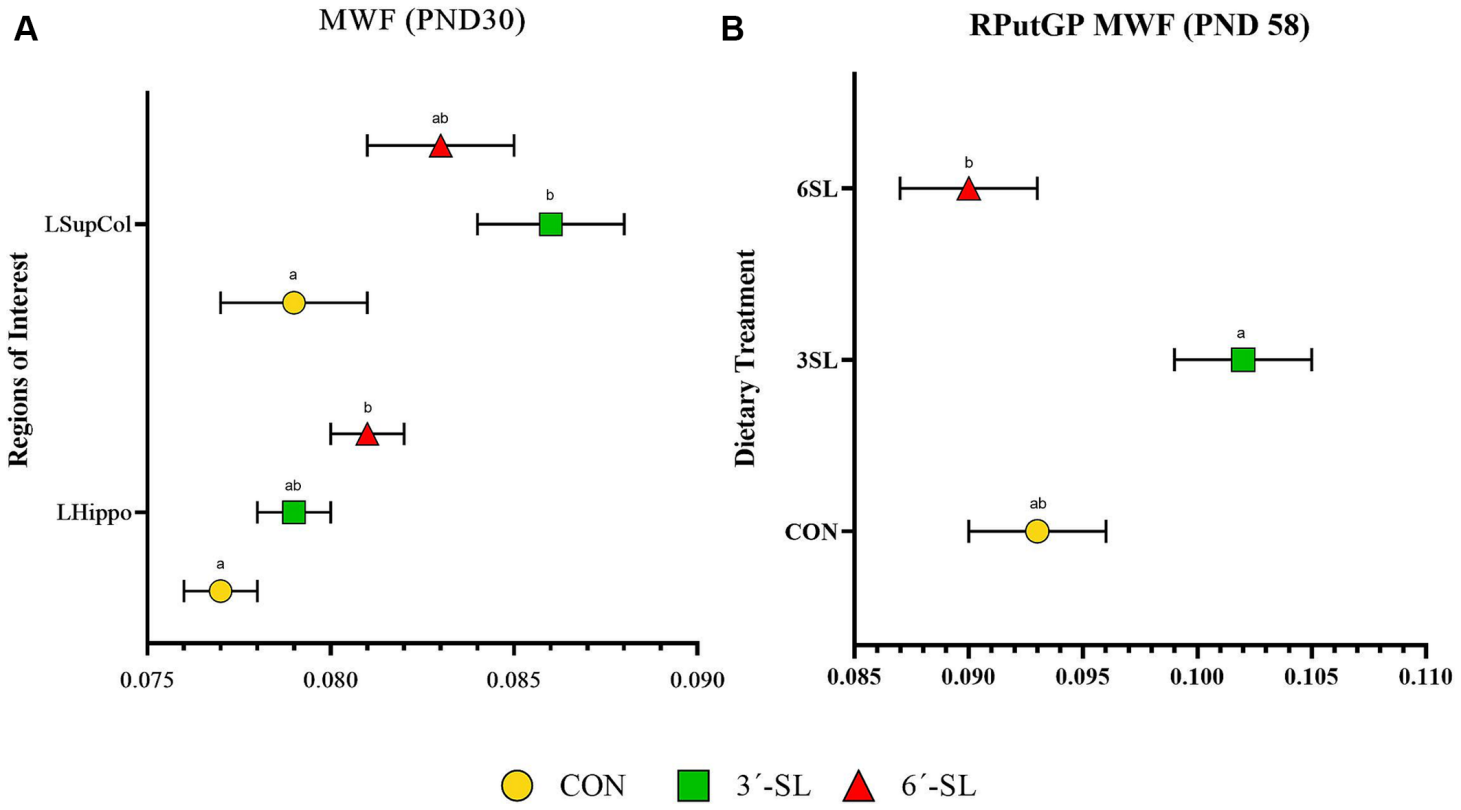
Myelin water fraction (MWF) outcomes in pigs on PND 30 and PND 58. **(A)** Myelin water fraction values at PND 30 are displayed for the left superior colliculus (LsupCol) and the left hippocampus (LHippo). **(B)** Myelin water fraction values for the right putamen-globus pallidus (RputGP) region at PND 58 are displayed. MWF, myelin water fraction; PND, postnatal day; CON, dietary group given a control diet; 3′-SL, dietary group supplemented with 0.273% 3′-S; 6′-SL, dietary group supplemented with 0.273% 6′-SL. ^ab^Means lacking a common superscript letter differ (*p* < 0.05).

Myelin water fraction values were calculated for the whole brain and across 26 distinct regions of interest (Figure 7; Table 3; Supplementary Table S13). Dietary differences in MWF values were observed at PND 30 in the left hippocampus and left superior colliculus (Figure 7). For the left hippocampus, MWF values in the 6′-SL group were higher (*p* = 0.023) compared with the CON group. For the left superior colliculus, MWF values in the 3′-SL group were higher (*p* = 0.06) compared with the CON group. Diet differences in MWF values at PND 58 were only observed in the right putamen/globus pallidus region (*p* = 0.030), where the 3′-SL group had larger values compared with the 6′-SL group.

**Table 3:** Myelin water fraction of pigs at PND 30.^1^

	Treatment			Pooled SEM	*p*-value
Region of interest	CON	3′-SL	6′-SL		
*n*	11	11	11	-	-
Whole brain	0.084	0.084	0.084	0.001	0.781
Combined cortex	0.083	0.083	0.083	0.001	0.979
Combined hippocampus	0.077	0.078	0.080	0.002	0.154
Combined internal capsule	0.116	0.113	0.116	0.002	0.588
Cerebellum	0.079	0.080	0.077	0.002	0.279
Corpus callosum	0.076	0.075	0.077	0.002	0.783
Hypothalamus	0.079	0.076	0.079	0.003	0.478
Left caudate	0.076	0.079	0.076	0.003	0.285
Left cortex	0.083	0.083	0.082	0.001	0.769
Left hippocampus	0.077^a^	0.079^ab^	0.081^b^	0.001	0.023
Left inferior colliculi	0.075	0.082	0.084	0.003	0.088
Left internal capsule	0.115	0.112	0.114	0.002	0.529
Left olfactory bulb	0.066	0.063	0.062	0.002	0.304
Left putamen-globus pallidus	0.095	0.091	0.096	0.003	0.341
Left superior colliculi	0.079^a^	0.086^b^	0.083^ab^	0.002	0.006
Medulla	0.075	0.075	0.075	0.001	0.968
Midbrain	0.084	0.083	0.082	0.002	0.338
Pons	0.089	0.090	0.086	0.001	0.123
Right caudate	0.078	0.077	0.081	0.002	0.187
Right cortex	0.082	0.082	0.083	0.001	0.952
Right hippocampus	0.077	0.079	0.081	0.002	0.220
Right inferior colliculi	0.079	0.079	0.072	0.003	0.150
Right internal capsule	0.118	0.115	0.117	0.002	0.664
Right olfactory bulb	0.064	0.061	0.060	0.002	0.103
Right putamen-globus pallidus	0.100	0.096	0.098	0.003	0.462
Right superior colliculi	0.083	0.083	0.081	0.002	0.774
Thalamus	0.099	0.098	0.099	0.002	0.987

3′-SL, 3′-sialyllactose; 6′-SL, 6′-sialyllactose; SEM, standard error of mean.

^ab^Means lacking a common superscript letter within a row differ (*p* < 0.05).

1 Data presented are least squares means and *p*-values from mixed model 1-way ANOVA.

## 4 Discussion

The study described herein expands on previous work from our lab exploring the influence of dietary SL supplementation in growing pigs. A paper by Fleming et al. (2018) explored the relationship between the addition of SL and cognitive ability, while Mudd et al. (2017) explored the influence of SL on structural brain development. The current study aimed to bridge the gap between these two contributions by directly observing the longitudinal impact of SL supplementation in the form of 3′-sialyllactose or 6′-sialyllactose on both cognitive and brain development in the same cohort of pigs. To our knowledge, this is the first study assessing the impact of consistent SL supplementation on both cognitive and brain development at multiple time-points when SL levels would typically fluctuate in human milk. The average concentration of sialyllactose in human milk is 500 mg/L (Martín-Sosa et al., 2004). This, along with previous pig SA supplementation work (Monaco et al., 2019, 2020), determined the concentration of SA used in this study. Cognitive development was assessed via the NOR task, while neuroimaging was utilized to assess structural and microstructural brain development. The impact of SL supplementation on cognitive development was minimal, as the CON pigs demonstrated similar learning to both supplementation groups at both time-points. The impact of SL supplementation on brain development is less clear, as results from both PND 33 and PND 61 indicate mixed effects among supplemented groups.

### 4.1 Behavior

In order to best merge the studies by Fleming et al. (2018) and Mudd et al. (2017), the NOR task in the current study followed the protocol used in Fleming et al. (2018). As such, the task utilized a 48-h delay period to test long-term memory, as opposed to shorter delays that test working memory. In accordance with Fleming et al. (2018), pigs in our study did not show behavioral differences across diet groups when performing the NOR task. This is in contrast to previous work in the field that has suggested supplementation of SA to young, growing pigs can benefit cognitive performance. This dichotomy may be explained by the source of the SA provided. Previous studies have supplemented SA-enriched sources, such as lactoferrin (Chen et al., 2015), casein glycomacropeptide (Wang et al., 2007b), or bovine milk oligosaccharide-enriched whey (Obelitz-Ryom et al., 2019), whereas our study supplemented essentially pure forms of 3′-SL and 6′-SL. Similarly, the type of task used to assess cognitive development may explain the differences in outcomes. The tasks utilized in previous studies tended to be operant conditioning-based (Wang et al., 2007b; Chen et al., 2015) or spatial-based (Obelitz-Ryom et al., 2019), which require different neural pathways than the NOR task that we employed. Operant conditioning tasks require rule learning (Poling et al., 2002; Nargeot and Puygrenier, 2019), while spatial tasks require locational awareness (Cole and Josselyn, 2008; Bird and Burgess, 2009) to perform the task appropriately. However, NOR relies on spontaneous, inherent behavior expressed by animals, such as the desire to explore novelty in their environment (Wood-Gush and Vestergaard, 1991; Dix and Aggleton, 1999). This difference in cognitive demand required for each task may be a reason cognitive differences were not seen in our study. It is possible that NOR does not require the neural pathways supported by the supplementation of SA in order to be performed.

While each cognitive task listed above requires a different neural pathway, ultimately triggering different brain regions in a different order, literature agrees that tasks requiring memory of any kind utilize the functionality of the hippocampus at some point in cognitive processing (Scoville and Milner, 1957; Eichenbaum, 2004). Recent efforts have been made to map the developmental trajectory of the whole brain and individual brain regions, including the hippocampus, in growing pigs. This research has found that overall, intact male pigs experience a whole-brain growth spurt later than females, with the growth spurt occurring just after 4 weeks of age in males. In addition to a whole-brain growth spurt just after 4 weeks, the male pig also experiences a region-specific growth spurt in the hippocampus around 8 weeks of age (Conrad et al., 2012). Behavioral testing at the first time-point in our study began on PND 24 with initiation of testing on PND 28. It is possible that our initial testing time-point was too early in the development of the male pig brain, specifically the hippocampus, to assess cognitive differences. Behavioral testing at the second time-point in our study began on PND 52 with testing on PND 56. Given that this timeframe is a critical period for hippocampal development, and therefore cognitive development, in male pigs, we would expect to see cognitive differences between diet groups if SA supplementation was influencing the neural pathways required for NOR.

Pigs supplemented with 3′-SL in the current study exhibited increased latency to the last interaction (i.e., interacted later in the trial) with either object than the 6′-SL pigs on PND 28. Similarly, the latency to the first sample object interaction was trending (*p* = 0.051) with CON pigs taking longer to interact on PND 28. These latency results coincide with previous pig supplementation work which also failed to show differences in RI outcomes but did show statistical differences in latency measures (Joung et al., 2020; Fil et al., 2021a; Sutkus et al., 2022). Rather than explaining cognitive differences, previous work has suggested that latency may just be an indicator of individual pig personality (Hessing et al., 1993). While they focused strictly on novelty, Hessing et al. (1993) suggested that pigs that were quick to interact with novelty were also quick to lose interest and deemed “proactive” pigs. Conversely, pigs that were slower to interact with novelty tended to interact longer with it and were deemed “reactive” pigs. Based on these findings, results from the current study suggest that 3′-SL and CON pigs may be classified as “reactive,” while 6′-SL pigs may be considered “proactive” on PND 28.

The effects of SA supplementation have also been measured in rodent studies utilizing the NOR task and are equally inconclusive. Weaned rats dosed with *N*-acetylmannosamine (ManAc), *N*-acetylglucosamine (GluAc), or *N*-acetylneuraminic acid (Neu5Ac) at a rate of 5.0 mg/mL showed no differences in recognition index when compared with a control group (Kikusui et al., 2012). The same study also investigated the impact of age on NOR performance and found that young rats demonstrated distinction between the familiar object and the novel object while middle-aged rats did not (Kikusui et al., 2012). Contradictory to the results of Kikusui et al. (2012), a study by Oliveros et al. (2018) investigating the impact of supplementing free Neu5Ac and 6′-SL during lactation employed the NOR task at both weaning (~PND 21) and 1 year of age but found no differences in cognitive performance at weaning age. However, both treatment groups preferred the novel object at 1 year of age, spending significantly more time with the novel object than the sample object, while the control group did not. This study also tested preference for novel location and found that both treatment groups, again, had preference for novelty only at 1 year of age while the control group did not (Oliveros et al., 2018).

### 4.2 Magnetic resonance imaging

In conjunction with Golden et al. (2023), the implications of SL supplementation on brain development were further assessed via anatomical, diffusion-weighted, and myelin-focused images acquired at both PND 30 and PND 58. These time-points have been identified as critical periods of brain development in the pig. Conrad et al. (2012) identified the 4-week time-point as the period of most rapid brain growth in the pig, indicating that this is an essential time for postnatal synaptogenesis and myelination (Rice and Barone, 2000; Jena et al., 2020). Hippocampal volume growth peaks around the 8-week time-point in male pigs (Conrad et al., 2012), at which point, the pig brain is approximately 80% of its full weight (Winick, 1969). Across all MRI results, time-points were analyzed separately due to a change in coil type: 8-channel head coil (PND 30) to an 18-channel flex body coil in combination with a 32-channel spine coil (PND 58). This switch was made due to increased head circumference at PND 58. While an increase in channel number for head coils is typically associated with gains in signal-to-noise ratio and spatial resolution (Parikh et al., 2011), the switch from a head coil to a body coil has the opposite effect. Therefore, neither treatment nor within-pig comparisons of results were considered across time.

Utilizing anatomical scans, absolute and relative volume were calculated for the whole brain, gray matter, white matter, cerebral spinal fluid, and 26 regions of interest at both PND 30 and PND 58. Although several diet effects were observed for absolute volume across various ROI at both time points, these differences mostly dissipated when the regional volumes were estimated relative to the whole brain volume of each pig. This indicates that allometric growth of the brain and corresponding regions was similar, signifying structural brain development was not likely altered by diet. Absolute volumes were quantified to account for differences in whole brain size across individual pigs. While relative volume across the majority of brain regions was not impacted by diet, the pons, a region that is part of the brain stem and plays a role in balance, was observed to have a larger relative volume in the 3′-SL group compared with the control diet at the 4-week time-point. Similar findings were reported by Sutkus et al. (2022), in which 4-week-old pigs supplemented with HMO had a larger relative volume in the pons compared with those not supplemented with HMO. On PND 58 in the current study, the 6′-SL group was observed to have a larger relative volume in both the cerebral aqueduct and midbrain. Despite this, relative volume results were minimal at both time-points. Similar results have been observed by Obelitz-Ryom et al. (2019), where pigs fed a diet enriched with a combination of 3′-SL and 6′-SL had similar structural volumes compared with pigs fed a control diet. Of note, the pigs utilized in this study were preterm and brain scanning was conducted *ex vivo* on PND 19 (Obelitz-Ryom et al., 2019). In another study, relative volumes across 19 separate brain regions in 4-week-old pigs were found to be largely unaltered by diets composed of a combination of prebiotics, lactoferrin, and milk fat globule membrane (Mudd et al., 2016a). In general, absolute and relative volumes of the whole brain have been commonly perceived as a metric of intelligence across species (Roth and Dicke, 2005). Although after further assessment, volume measurements have been determined to be poor measures of intelligence, as body size of the animal is characterized as a “confounding variable” and even when corrected, species inconsistencies persist (Roth and Dicke, 2005). Therefore, intelligence is better characterized by information processing capacity which is reflected in tissue cellular composition and neuron packing density across the brain (Roth and Dicke, 2005). To identify these features, investigation of microstructure organization and myelination are of interest.

Diffusion tensor imaging is an imaging technique that provides insight into fiber structure organization by assessing 4 subsets of water diffusion across the brain: axial diffusivity (AD), radial diffusivity (RD), mean diffusivity (MD), and fractional anisotropy (FA) (Alexander et al., 2007). AD and RD provide insight into fiber integrity by assessing how water molecules move parallel or perpendicular to fiber tracts, respectively (Alexander et al., 2007; Bennett et al., 2010). While increased RD levels have been associated with myelination issues, the same is not necessarily true for AD values (Alexander et al., 2007). Changes in AD values have been identified to be more specific to axonal damage and loss (Alexander et al., 2007). Most research in early-life brain development is dedicated to assessing MD and FA values. MD is referred to as the apparent diffusion coefficient, or average diffusivity in the brain, whereas FA corresponds to the degree that water molecules are flowing in a particular orientation across the brain (Alexander et al., 2007). It is understood that FA values continuously increase with age, specifically during periods of brain development (Barnea-Goraly et al., 2005; Qi et al., 2017). Additionally, FA has been associated with white matter integrity, or the degree of myelination in the brain (Moseley et al., 1990; Pierpaoli et al., 1996). Although this association has been widely studied, many additional factors can alter FA values, such as the crossing of white matter fibers (Alexander et al., 2007). As such, continued efforts to better understand the relationship between myelination and white matter integrity are needed.

In the present study, there were no treatment effects across diffusion outcomes at PND 30 except for FA. FA values for overall white matter and some white matter-enriched regions were higher in control pigs compared with both SL supplemented groups. This is contrary to previous work by Mudd et al. (2017) who found altered AD, MD, and RD values in SL supplemented groups and no differences in FA. In this previous work, SL was supplemented at varying dosages and researchers observed that the treatment group supplemented with 380 mg SL/L (i.e., a moderate dose) displayed the most diffusion differences, while other treatment groups did not differ from the control group. Notably, the treatment group with higher levels of SL (760 mg SL/L) displayed diffusion values across all regions like those of the control group. In the present study, SL was supplemented at a dose of 500 mg SL/L which is higher than the moderate dose in the Mudd et al. (2017) paper and may be an explanation for the lack of treatment differences observed, thereby reinforcing the presence of an upper threshold. Additionally, since there was an image acquisition issue that caused data to be unstable on PND 30, the overall sample size for each treatment group was greatly reduced. With a higher sample size, differences may have been observed. However, the current sample size was comparable to Mudd et al. (2017).

On PND 58, several diffusivity differences across treatment groups were detected. AD, RD, and MD values were observed to be lower in the left hippocampus for the 6′-SL group compared with the control group, and AD and MD were observed to be lower in the cerebellum for the control group compared with either treatment groups. Throughout brain development, diffusion frequently fluctuates, lowering the water content of white matter when compared with gray matter (Hüppi and Dubois, 2006; Dubois et al., 2014). As the brain matures, changes in diffusivity values are expected as more structures, including cells and axonal membranes, and fibers begin to hinder the motion of water molecules (Hüppi and Dubois, 2006). Therefore, during white matter development, decreases in overall diffusion (i.e., MD) and RD are expected (Hüppi and Dubois, 2006). This concept was supported in a study utilizing young pigs, specifically when altering choline status during prenatal and postnatal growth periods (Mudd et al., 2016b). In pigs receiving a choline-sufficient diet, MD and RD values, specifically in the cerebellum, were found to be lower than in choline-deficient pigs (Mudd et al., 2016b). Contrary to previous research (Qi et al., 2017), the current study observed that groups supplemented with SL had decreased FA values in the right hippocampus and thalamus compared with the control group. Typically, as previously mentioned, FA values increase with age, indicating brain maturation (Barnea-Goraly et al., 2005; Hüppi and Dubois, 2006). In several studies utilizing pigs, diffusion was found to be largely unaffected by SL supplementation alone (Mudd et al., 2017; Obelitz-Ryom et al., 2019). Overall, it is difficult to truly assess what may have caused lower FA values over time, as this is the first study, to our knowledge, to assess prolonged SL supplementation. It is possible that these diffusivity values are not absolutely related to age-related changes in microstructural organization, and further studies are warranted to elucidate variability in diffusivity measures.

To assess changes more accurately in myelination within the brain, myelin water imaging is utilized. This imaging technique isolates distinct water pools within the myelin sheath that wraps around axons (Dubois et al., 2014). Water molecules that are either free-floating in extracellular space or trapped within the lipid bilayer of the myelin sheath emit distinct signatures that can be captured by magnetic resonance signals to calculate myelin water fraction (MWF) (Dubois et al., 2014; Lee et al., 2021). MWF has been identified as a biomarker and reflection of myelin concentration (Lee et al., 2021). Across early development and into adulthood, myelination of white matter fibers is a critical step for developing efficient brain networks (Dubois et al., 2014). As such, increases in myelination are associated with improved cognition and brain maturation (Gibson, 1991). Myelination first occurs in areas such as the brainstem and then moves outward towards the cerebral hemispheres (Gibson, 1991). Although typically similar across the whole brain and most regions, in the present study, MWF was observed to be higher in the left hippocampus for the 6′-SL supplemented group. It is well understood that the hippocampus is continuously myelinating from birth to adulthood (Benes, 1989), meaning that 6′-SL may have accentuated this developmental change. However, it is important to note that these increases were only observed in the left hippocampus, and when evaluating changes to the combined hippocampus, treatment effects dissipated. Additionally, MWF values in the left superior colliculus, a region important for combining eye and head movements (Zubricky and Das, 2022), were observed to be higher in pigs supplemented with 3′-SL compared with the control group. Again, this was only observed in one hemisphere, possibly indicating an inconsistent response. Although these effects were only observed unilaterally, hemispheric differences with sialic acid concentrations have been noted previously. Wang et al. (1998) found that the left lobe of the chimpanzee brain had higher concentration of SA compared with the right, however, these differences were observed at 2 years of age. Since pigs are a rapidly developing species (Conrad et al., 2012), it is possible that these hemispheric differences are detected earlier, although further research is necessary to confirm this hypothesis. MWF values on PND 58 were similar across treatment groups, except for the right putamen and globus pallidus, where the 3′-SL group was observed to have higher values than the 6′-SL group. These regions are important for proprioceptive movements and essential brain functions (Ghandili and Munakomi, 2023; Javed and Cascella, 2023). Similar findings have been reported by Fil et al. (2021a), where no differences in MWF were observed between dietary treatments across multiple time-points. The findings from the current study suggest similar brain development, regardless of dietary treatment.

Sialylated HMO have been associated with brain and cognitive development as they function as an important exogenous source of SA for the growing infant (Liu et al., 2022). Although the body can synthesize SA via *de novo* synthesis, previous research has alluded to a majority of early-life SA concentrations originating from exogenous sources, such as SL (Karim and Wang, 2006; Wang, 2009; Liu et al., 2022). Although the mechanism behind digestion and absorption of sialyllactose remains unclear, SA is believed to be liberated from the sialyllactose chain after fermentation in the colon (Karim and Wang, 2006). Several studies have also found increased concentrations of SA in the brain several hours after intravenous administration of free sialic acid in rodents and piglets, alluding to a mechanism of SA absorption in the brain (Nöhle et al., 1982; Karim and Wang, 2006; Wang et al., 2007a). Furthermore, SA has been classified as an important nutrient for brain growth due to its relatively high concentration in the brain, as well as the fundamental role it plays in the central nervous system (Liu et al., 2022). Sialic acid molecules can combine to form polysialic acid, which is a main component of the neural cell adhesion molecule (NCAM) (Hildebrandt et al., 2007; Wang, 2009; Sato and Kitajima, 2019; Liu et al., 2022). NCAM is critical in nervous system development as it plays roles in neurogenesis, synaptic formation, and memory formation (Hildebrandt et al., 2007; Liu et al., 2022). Rodent research has highlighted neurological impairments (e.g., impaired synaptic activity in the hippocampus) associated with lower concentrations of polysialic acid (Sato and Kitajima, 2019) However, there is still debate as to the importance of SA, as other rodent research testing the supplementation of SL observed no differences in brain SA-related gene expression (Duncan et al., 2009). Therefore, due to the downstream and cellular-structural impacts of SA, a potential limitation of the current study is the sensitivity by which brain development was quantified through noninvasive techniques.

It is possible that the neuroimaging and cognitive procedures utilized did not accurately detect microstructural changes caused by SL supplementation. Future research should capture changes of cellular structure through histological analysis and potentially utilize alternative cognitive paradigms. Also of note, the control diet for the current study contained whey protein, which may contain SL (Barile et al., 2009). Although diet formulations were designed to account for background SL, future studies should consider a control diet devoid of any SA or SL. It is also known that human brains contain significantly higher concentrations of SA than other species, and pertinent to the current study, about 4 times the concentration of SA found in the pig brain (Wang et al., 1998). Differences in brain development due to brain SA concentrations may be more apparent in human studies due to differences between humans and pigs. Similarly, developmental differences between boars and gilts may have an influence on both SA concentrations as well as activation of SA. Future studies are warranted to elucidate these differences. While this is the first study, to our knowledge, to assess both structural and cognitive changes due to longitudinal SL supplementation in a preclinical model, future investigations are warranted to elucidate the relationship between dietary SL or SA supplementation and cognitive development.

## 5 Conclusion

In summary, we investigated the impacts of longitudinal 3′-SL or 6′-SL supplementation on cognitive and structural brain development in pigs. Similar cognitive and structural development across treatments indicate a lack of harm from the supplementation. Using the NOR task and neuroimaging techniques, brain development was assessed at both PND 33 and PND 61. Regardless of dietary treatment, pigs performed the NOR task similarly at both time-points alluding to similar cognitive development. Across three neuroimaging modalities, minimal diet-specific differences were observed, which may indicate structural changes that are more apparent at microstructural levels. Although minimal differences were found, this research provides a base for future work to investigate HMO supplementation with a goal of supporting cognitive and brain development through early-life nutrition.

## Data availability statement

The datasets presented in this article are not readily available because commercial products were tested. Requests to access the datasets should be directed to RD, rdilger2@illinois.edu.

## Ethics statement

The animal study was approved by Institutional Animal Care and Use Committee (IACUC), University of Illinois at Urbana-Champaign. The study was conducted in accordance with the local legislation and institutional requirements.

## Author contributions

RG: Data curation, Formal analysis, Investigation, Methodology, Visualization, Writing – original draft, Writing – review & editing. LS: Data curation, Formal analysis, Investigation, Methodology, Visualization, Writing – original draft, Writing – review & editing. SD: Conceptualization, Formal analysis, Funding acquisition, Investigation, Project administration, Resources, Supervision, Writing – review & editing. RD: Conceptualization, Data curation, Formal analysis, Funding acquisition, Investigation, Methodology, Project administration, Resources, Software, Supervision, Visualization, Writing – original draft, Writing – review & editing.

## Footnotes

^1^https://fsl.fmrib.ox.ac.uk/fslcourse/2019_Beijing/lectures/FDT/fdt1.html
