# Supplementary material for: Dietary supplementation of 3′-sialyllactose or 6′-sialyllactose elicits minimal influence on cognitive and brain development in growing pigs
Source: Front Behav Neurosci. 2024 Jan 10;17:1337897. doi: 10.3389/fnbeh.2023.1337897 (PMC10806065; doi:10.3389/fnbeh.2023.1337897)
Supplement: Supplementary file 1 [file Data_Sheet_1.docx]

| **Supplemental Table 1.** Absolute brain volumes (mm^3^) of pigs at PND 30^1^ | | | | | |
| --- | --- | --- | --- | --- | --- |
|  | **Diet** | | | **Pooled SEM** | ***P*-value** |
| **ROI** | **CON** | **3ˊ-SL** | **6ˊ-SL** |  |  |
| *n* | 11 | 12 | 11 | - | - |
| Whole brain | 62,664 | 60,421 | 59,872 | 1,125 | 0.137 |
| Grey matter | 35,363 | 35,259 | 33,985 | 1,049 | 0.374 |
| White matter | 18,632^a^ | 17,495^b^ | 17,905^ab^ | 547.9 | **0.043** |
| Cerebral spinal fluid | 8,689 | 7,737 | 7,719 | 785.5 | 0.189 |
| Cerebellum | 8,345 | 8,271 | 7,999 | 191.1 | 0.372 |
| Cerebral aqueduct | 57.3 | 56.2 | 55.8 | 1.13 | 0.502 |
| Corpus callosum | 592.0^a^ | 559.5^ab^ | 533.0^b^ | 13.47 | **0.018** |
| Fourth ventricle | 222.5 | 216.7 | 219.0 | 6.87 | 0.745 |
| Hypothalamus | 203.8 | 194.2 | 195.9 | 3.40 | 0.130 |
| Lateral ventricle | 827.4^a^ | 785.1^ab^ | 749.0^b^ | 19.38 | **0.026** |
| Left caudate | 425.3^a^ | 405.1^ab^ | 384.3^b^ | 10.28 | **0.029** |
| Left cortex | 21,642 | 21,058 | 20,745 | 467.1 | 0.366 |
| Left hippocampus | 556.3 | 533.6 | 525.5 | 11.35 | 0.098 |
| Left inferior colliculi | 149.2 | 146.4 | 147.1 | 2.60 | 0.565 |
| Left internal capsule | 932.2^a^ | 884.4^ab^ | 853.1^b^ | 19.82 | **0.029** |
| Left olfactory bulb | 1,727 | 1,738 | 1,767 | 72.53 | 0.922 |
| Left putamen-globus pallidus | 272.5^a^ | 258.7^ab^ | 248.9^b^ | 6.31 | **0.041** |
| Left superior colliculi | 299.0 | 284.5 | 292.6 | 5.51 | 0.092 |
| Medulla | 2,299 | 2,240 | 2,234 | 55.45 | 0.623 |
| Midbrain | 2,817 | 2,723 | 2,759 | 45.71 | 0.189 |
| Pons | 1,804 | 1,820 | 1,775 | 27.54 | 0.382 |
| Right caudate | 399.4^a^ | 379.8^ab^ | 363.6^b^ | 8.56 | **0.024** |
| Right cortex | 21,248 | 20,834 | 20,348 | 406.5 | 0.289 |
| Right hippocampus | 598.5 | 578.8 | 566.1 | 11.34 | 0.111 |
| Right inferior colliculi | 142.5 | 141.4 | 141.5 | 2.79 | 0.903 |
| Right internal capsule | 986.7^a^ | 945.6^ab^ | 913.6^b^ | 15.64 | **0.014** |
| Right olfactory bulb | 1,880 | 1,827 | 1,874 | 70.33 | 0.813 |
| Right putamen-globus pallidus | 288.9^a^ | 277.8^ab^ | 266.8^b^ | 4.74 | **0.013** |
| Right superior colliculi | 288.0 | 278.8 | 284.8 | 5.57 | 0.298 |
| Thalamus | 1,663^a^ | 1,574^b^ | 1,551^b^ | 23.61 | **0.009** |
| ^1^Data presented are least squares means and *P*-values from mixed model 1-way ANOVA.  Abbreviations: PND, postnatal day; CON, control; 3ˊ-SL, 3ˊ-sialyllactose; 6ˊ-SL, 6ˊ-sialyllactose; ROI, regions of interest; SEM, standard error of mean.  ^ab^Means lacking a common superscript letter within a row differ (*P* < 0.05). | | | | | |

| **Supplemental Table 2.** Relative brain volumes (% of total brain volume) pigs at PND 30^1^ | | | | | |
| --- | --- | --- | --- | --- | --- |
|  | **Diet** | | | **Pooled SEM** | ***P*-value** |
| **ROI** | **CON** | **3ˊ-SL** | **6ˊ-SL** |  |  |
| *n* | 11 | 12 | 11 | - | - |
| Grey matter | 56.38 | 58.06 | 56.83 | 1.604 | 0.175 |
| White matter | 29.80 | 28.87 | 29.72 | 0.784 | 0.311 |
| Cerebral spinal fluid | 13.76 | 12.66 | 13.76 | 1.188 | 0.470 |
| Cerebellum | 13.32 | 13.70 | 13.34 | 0.263 | 0.258 |
| Cerebral aqueduct | 0.09 | 0.09 | 0.09 | 0.001 | 0.478 |
| Corpus callosum | 0.94 | 0.93 | 0.89 | 0.022 | 0.205 |
| Fourth ventricle | 0.35 | 0.36 | 0.36 | 0.012 | 0.447 |
| Hypothalamus | 0.32 | 0.32 | 0.33 | 0.005 | 0.445 |
| Lateral ventricle | 1.32 | 1.30 | 1.26 | 0.030 | 0.257 |
| Left caudate | 0.68 | 0.67 | 0.64 | 0.016 | 0.226 |
| Left cortex | 34.61 | 34.92 | 34.65 | 0.500 | 0.858 |
| Left hippocampus | 0.89 | 0.87 | 0.88 | 0.015 | 0.404 |
| Left inferior colliculi | 0.24 | 0.24 | 0.25 | 0.003 | 0.418 |
| Left internal capsule | 1.48 | 1.46 | 1.42 | 0.028 | 0.155 |
| Left olfactory bulb | 2.74 | 2.87 | 2.97 | 0.104 | 0.335 |
| Left putamen-globus pallidus | 0.43 | 0.43 | 0.41 | 0.010 | 0.205 |
| Left superior colliculi | 0.48 | 0.47 | 0.49 | 0.007 | 0.449 |
| Medulla | 3.65 | 3.71 | 3.73 | 0.102 | 0.466 |
| Midbrain | 4.51 | 4.52 | 4.61 | 0.047 | 0.228 |
| Pons | 2.92^a^ | 3.02^b^ | 2.97^ab^ | 0.037 | **0.050** |
| Right caudate | 0.64 | 0.63 | 0.61 | 0.013 | 0.331 |
| Right cortex | 33.92 | 34.52 | 34.01 | 0.505 | 0.484 |
| Right hippocampus | 0.96 | 0.96 | 0.94 | 0.016 | 0.617 |
| Right inferior colliculi | 0.23 | 0.23 | 0.24 | 0.004 | 0.185 |
| Right internal capsule | 1.57 | 1.57 | 1.54 | 0.027 | 0.404 |
| Right olfactory bulb | 3.00 | 3.03 | 3.14 | 0.099 | 0.572 |
| Right putamen-globus pallidus | 0.46 | 0.47 | 0.45 | 0.011 | 0.169 |
| Right superior colliculi | 0.46 | 0.46 | 0.47 | 0.007 | 0.323 |
| Thalamus | 2.65 | 2.61 | 2.60 | 0.032 | 0.331 |
| ^1^Data presented are least squares means and *P*-values from mixed model 1-way ANOVA.  Abbreviations: PND, postnatal day; CON, control; 3ˊ-SL, 3ˊ-sialyllactose; 6ˊ-SL, 6ˊ-sialyllactose; ROI, regions of interest; SEM, standard error of mean.  ^ab^Means lacking a common superscript letter within a row differ (*P* < 0.05). | | | | | |

| **Supplemental Table 3.** Absolute brain volumes (mm^3^) of pigs at PND 58^1^ | | | | | |
| --- | --- | --- | --- | --- | --- |
|  | **Diet** | | | **Pooled SEM** | ***P*-value** |
| **ROI** | **CON** | **3ˊ-SL** | **6ˊ-SL** |  |  |
| *n* | 11 | 12 | 11 | - | - |
| Whole brain | 86,604 | 84,083 | 82,151 | 1,555 | 0.093 |
| Grey matter | 34,899 | 32,683 | 34,127 | 1,261 | 0.316 |
| White matter | 31,705 | 30,393 | 32,042 | 1,181 | 0.564 |
| Cerebral spinal fluid | 11,227 | 11,021 | 11,067 | 635.6 | 0.960 |
| Cerebellum | 9,521 | 9,428 | 9,341.3 | 235.0 | 0.828 |
| Cerebral aqueduct | 29.7 | 28.5 | 29.6 | 0.59 | 0.156 |
| Corpus callosum | 590.9^a^ | 565.4^ab^ | 532.7^b^ | 14.72 | **0.037** |
| Fourth ventricle | 71.5 | 67.1 | 69.9 | 2.44 | 0.356 |
| Hypothalamus | 201.7 | 192.6 | 198.6 | 4.32 | 0.255 |
| Lateral ventricle | 704.4^a^ | 675.0^ab^ | 631.3^b^ | 17.44 | **0.026** |
| Left caudate | 382.0^a^ | 362.6^ab^ | 346.9^b^ | 8.43 | **0.021** |
| Left cortex | 27,095 | 26,444 | 26,197 | 614.5 | 0.497 |
| Left hippocampus | 544.3 | 529.1 | 509.0 | 12.35 | 0.077 |
| Left inferior colliculi | 138.6 | 138.7 | 136.8 | 2.27 | 0.673 |
| Left internal capsule | 982.9 | 955.4 | 928.9 | 18.14 | 0.116 |
| Left olfactory bulb | 1,755 | 2,055 | 1,867 | 224.9 | 0.533 |
| Left putamen-globus pallidus | 247.3 | 237.7 | 233.2 | 5.29 | 0.130 |
| Left superior colliculi | 294.2 | 288.2 | 290.5 | 5.72 | 0.631 |
| Medulla | 2,732 | 2,622 | 2,729 | 72.27 | 0.398 |
| Midbrain | 3,349 | 3,251 | 3,304 | 52.42 | 0.176 |
| Pons | 2,162 | 2,102 | 2,144 | 47.22 | 0.394 |
| Right caudate | 389.8^a^ | 375.9^ab^ | 356.7^b^ | 8.89 | **0.038** |
| Right cortex | 26,127 | 25,366 | 25,435 | 622.7 | 0.539 |
| Right hippocampus | 536.0 | 530.6 | 519.9 | 11.87 | 0.546 |
| Right inferior colliculi | 143.0 | 140.0 | 142.1 | 2.48 | 0.422 |
| Right internal capsule | 912.4 | 865.4 | 854.5 | 17.41 | 0.052 |
| Right olfactory bulb | 1,642 | 1,836 | 1,620 | 207.0 | 0.679 |
| Right putamen-globus pallidus | 225.4 | 218.5 | 211.8 | 4.86 | 0.139 |
| Right superior colliculi | 301.4 | 298.2 | 298.1 | 6.52 | 0.895 |
| Thalamus | 1,678^a^ | 1,588^b^ | 1,543^b^ | 30.92 | **0.018** |
| ^1^Data presented are least squares means and *P*-values from mixed model 1-way ANOVA.  Abbreviations: PND, postnatal day; CON, control; 3ˊ-SL, 3ˊ-sialyllactose; 6ˊ-SL, 6ˊ-sialyllactose; ROI, regions of interest; SEM, standard error of mean.  ^ab^Means lacking a common superscript letter within a row differ (*P* < 0.05). | | | | | |

| **Supplemental Table 4.** Relative brain volumes (% of total brain volume) of pigs at PND 58^1^ | | | | | |
| --- | --- | --- | --- | --- | --- |
|  | **Diet** | | | **Pooled SEM** | ***P*-value** |
| **ROI** | **CON** | **3ˊ-SL** | **6ˊ-SL** |  |  |
| *n* | 11 | 12 | 11 | - | - |
| Grey matter | 40.62 | 39.06 | 41.30 | 1.349 | 0.374 |
| White matter | 36.48 | 36.23 | 39.02 | 1.391 | 0.312 |
| Cerebral spinal fluid | 12.89 | 13.09 | 13.52 | 0.719 | 0.762 |
| Cerebellum | 11.04 | 11.23 | 11.37 | 0.208 | 0.403 |
| Cerebral aqueduct | 0.034^a^ | 0.034^a^ | 0.036^b^ | 0.001 | **0.021** |
| Corpus callosum | 0.68 | 0.66 | 0.65 | 0.011 | 0.150 |
| Fourth ventricle | 0.08 | 0.08 | 0.09 | 0.003 | 0.277 |
| Hypothalamus | 0.23 | 0.23 | 0.24 | 0.005 | 0.071 |
| Lateral ventricle | 0.81 | 0.78 | 0.77 | 0.014 | 0.119 |
| Left caudate | 0.44 | 0.43 | 0.42 | 0.007 | 0.185 |
| Left cortex | 31.27 | 31.33 | 31.68 | 0.347 | 0.663 |
| Left hippocampus | 0.63 | 0.62 | 0.62 | 0.009 | 0.585 |
| Left inferior colliculi | 0.16 | 0.17 | 0.17 | 0.002 | 0.239 |
| Left internal capsule | 1.15 | 1.12 | 1.14 | 0.013 | 0.208 |
| Left olfactory bulb | 2.00 | 2.43 | 2.27 | 0.258 | 0.388 |
| Left putamen-globus pallidus | 0.29 | 0.28 | 0.28 | 0.004 | 0.888 |
| Left superior colliculi | 0.34 | 0.34 | 0.35 | 0.006 | 0.387 |
| Medulla | 3.14 | 3.12 | 3.32 | 0.075 | 0.156 |
| Midbrain | 3.89^a^ | 3.87^a^ | 4.02^b^ | 0.037 | **0.015** |
| Pons | 2.49 | 2.50 | 2.59 | 0.046 | 0.192 |
| Right caudate | 0.45 | 0.45 | 0.43 | 0.008 | 0.366 |
| Right cortex | 30.14 | 30.08 | 30.75 | 0.390 | 0.411 |
| Right hippocampus | 0.62 | 0.63 | 0.63 | 0.010 | 0.722 |
| Right inferior colliculi | 0.17 | 0.17 | 0.17 | 0.002 | 0.160 |
| Right internal capsule | 1.05 | 1.03 | 1.04 | 0.016 | 0.578 |
| Right olfactory bulb | 1.85 | 2.17 | 1.96 | 0.231 | 0.575 |
| Right putamen-globus pallidus | 0.26 | 0.26 | 0.26 | 0.005 | 0.951 |
| Right superior colliculi | 0.35 | 0.36 | 0.36 | 0.006 | 0.294 |
| Thalamus | 1.93 | 1.86 | 1.89 | 0.025 | 0.126 |
| ^1^Data presented are least squares means and *P*-values from mixed model 1-way ANOVA.  Abbreviations: PND, postnatal day; CON, control; 3ˊ-SL, 3ˊ-sialyllactose; 6ˊ-SL, 6ˊ-sialyllactose; ROI, regions of interest; SEM, standard error of mean.  ^ab^Means lacking a common superscript letter within a row differ (*P* < 0.05). | | | | | |

| Supplemental Table 5. Axial diffusivity values (AD; x 10⁻³ /mm²/s) of pigs at PND 30^1^ | | | | | |
| --- | --- | --- | --- | --- | --- |
|  | **Diet** | | | **Pooled SEM** | ***P*-value** |
| Region of Interest | **CON** | **3ˊ-SL** | **6ˊ-SL** |  |  |
| *n* | 6 | 7 | 8 | **-** | **-** |
| Cerebellum | 0.628 | 0.607 | 0.595 | 0.012 | 0.063 |
| Corpus callosum | 0.781 | 0.782 | 0.787 | 0.014 | 0.927 |
| Left caudate | 0.793 | 0.777 | 0.797 | 0.015 | 0.571 |
| Left hippocampus | 0.799 | 0.805 | 0.813 | 0.022 | 0.708 |
| Left internal capsule | 0.768 | 0.755 | 0.769 | 0.012 | 0.535 |
| Left cortex | 0.791 | 0.777 | 0.788 | 0.013 | 0.634 |
| Right caudate | 0.790 | 0.779 | 0.797 | 0.017 | 0.721 |
| Right hippocampus | 0.788 | 0.783 | 0.806 | 0.017 | 0.546 |
| Right internal capsule | 0.758 | 0.754 | 0.764 | 0.013 | 0.828 |
| Right cortex | 0.777 | 0.771 | 0.785 | 0.012 | 0.610 |
| Thalamus | 0.719 | 0.712 | 0.724 | 0.012 | 0.725 |
| White matter | 0.713 | 0.709 | 0.716 | 0.010 | 0.826 |
| Average AD mask | 0.651 | 0.656 | 0.667 | 0.018 | 0.587 |
| ^1^Data presented are least squares means and *P*-values from mixed model 1-way ANOVA. Abbreviations: PND, postnatal day; CON; control; 3ˊ-SL, 3ˊ-sialyllactose; 6ˊ-SL, 6ˊ-sialyllactose; SEM, standard error of mean. | | | | | |

| Supplemental Table 6. Radial diffusivity values (RD; x 10⁻³ /mm²/s) of pigs at PND 30^1^ | | | | | |
| --- | --- | --- | --- | --- | --- |
|  | **Diet** | | | **Pooled SEM** | ***P*-value** |
| Region of Interest | **CON** | **3ˊ-SL** | **6ˊ-SL** |  |  |
| *n* | 6 | 7 | 8 | **-** | **-** |
| Cerebellum | 0.399 | 0.386 | 0.381 | 0.007 | 0.094 |
| Corpus callosum | 0.485 | 0.492 | 0.495 | 0.010 | 0.767 |
| Left caudate | 0.449 | 0.440 | 0.454 | 0.010 | 0.551 |
| Left hippocampus | 0.462 | 0.470 | 0.475 | 0.015 | 0.608 |
| Left internal capsule | 0.356 | 0.359 | 0.369 | 0.007 | 0.182 |
| Left cortex | 0.497 | 0.495 | 0.501 | 0.008 | 0.693 |
| Right caudate | 0.465 | 0.470 | 0.476 | 0.010 | 0.736 |
| Right hippocampus | 0.462 | 0.467 | 0.473 | 0.011 | 0.667 |
| Right internal capsule | 0.358 | 0.359 | 0.364 | 0.007 | 0.794 |
| Right cortex | 0.485 | 0.488 | 0.496 | 0.006 | 0.431 |
| Thalamus | 0.422 | 0.415 | 0.423 | 0.006 | 0.528 |
| White matter | 0.409 | 0.406 | 0.412 | 0.005 | 0.518 |
| Average RD mask | 0.398 | 0.403 | 0.410 | 0.010 | 0.427 |
| ^1^Data presented are least squares means and *P*-values from mixed model 1-way ANOVA. Abbreviations: PND, postnatal day; CON; control; 3ˊ-SL, 3ˊ-sialyllactose; 6ˊ-SL, 6ˊ-sialyllactose; SEM, standard error of mean. | | | | | |

| Supplemental Table 7. Mean diffusivity values (MD; x 10⁻³ /mm²/s) of pigs at PND 30^1^ | | | | | |
| --- | --- | --- | --- | --- | --- |
|  | **Treatment** | | | **Pooled SEM** | ***P*-value** |
| Region of Interest | **CON** | **3ˊ-SL** | **6ˊ-SL** |  |  |
| *n* | 6 | 7 | 8 | **-** | **-** |
| Cerebellum | 0.475 | 0.460 | 0.452 | 0.009 | 0.078 |
| Corpus callosum | 0.584 | 0.588 | 0.593 | 0.011 | 0.839 |
| Left caudate | 0.562 | 0.553 | 0.569 | 0.011 | 0.553 |
| Left hippocampus | 0.575 | 0.581 | 0.587 | 0.017 | 0.629 |
| Left internal capsule | 0.501 | 0.491 | 0.502 | 0.007 | 0.421 |
| Left cortex | 0.595 | 0.589 | 0.597 | 0.009 | 0.717 |
| Right caudate | 0.574 | 0.573 | 0.583 | 0.012 | 0.772 |
| Right hippocampus | 0.570 | 0.572 | 0.584 | 0.013 | 0.609 |
| Right internal capsule | 0.505 | 0.490 | 0.498 | 0.006 | 0.237 |
| Right cortex | 0.582 | 0.582 | 0.592 | 0.008 | 0.526 |
| Thalamus | 0.520 | 0.514 | 0.523 | 0.008 | 0.601 |
| White matter | 0.510 | 0.507 | 0.514 | 0.007 | 0.680 |
| Average MD mask | 0.483 | 0.487 | 0.495 | 0.013 | 0.501 |
| ^1^Data presented are least squares means and *P*-values from mixed model 1-way ANOVA. Abbreviations: PND, postnatal day; CON; control; 3ˊ-SL, 3ˊ-sialyllactose; 6ˊ-SL, 6ˊ-sialyllactose; SEM, standard error of mean. | | | | | |

| Supplemental Table 8. Fractional anisotropy values (FA; arbitrary units) of pigs at PND 30^1^ | | | | | |
| --- | --- | --- | --- | --- | --- |
|  | **Treatment** | | | **Pooled SEM** | ***P*-value** |
| Region of Interest | **CON** | **3ˊ-SL** | **6ˊ-SL** |  |  |
| *n* | 11 | 11 | 11 | **-** | **-** |
| Cerebellum | 0.294^a^ | 0.293^a^ | 0.289^b^ | 0.001 | **0.005** |
| Corpus callosum | 0.317 | 0.311 | 0.311 | 0.005 | 0.691 |
| Left caudate | 0.363 | 0.357 | 0.356 | 0.005 | 0.506 |
| Left hippocampus | 0.346 | 0.340 | 0.341 | 0.007 | 0.733 |
| Left internal capsule | 0.476 | 0.460 | 0.455 | 0.007 | 0.055 |
| Left cortex | 0.302 | 0.295 | 0.295 | 0.002 | 0.054 |
| Right caudate | 0.337 | 0.327 | 0.331 | 0.006 | 0.521 |
| Right hippocampus | 0.326 | 0.324 | 0.331 | 0.006 | 0.618 |
| Right internal capsule | 0.479^a^ | 0.461^b^ | 0.459^b^ | 0.006 | **0.030** |
| Right cortex | 0.306 | 0.301 | 0.302 | 0.003 | 0.347 |
| Thalamus | 0.348 | 0.352 | 0.349 | 0.005 | 0.771 |
| White matter | 0.362^a^ | 0.355^b^ | 0.352^b^ | 0.003 | **0.047** |
| Average FA mask | 0.322 | 0.320 | 0.319 | 0.002 | 0.448 |
| ^1^Data presented are least squares means and *P*-values from mixed model 1-way ANOVA. Abbreviations: PND, postnatal day; CON; control; 3ˊ-SL, 3ˊ-sialyllactose; 6ˊ-SL, 6ˊ-sialyllactose; SEM, standard error of mean. | | | | | |

| Supplemental Table 9. Axial diffusivity values (AD; x 10⁻³ /mm²/s) of pigs at PND 58^1^ | | | | | |
| --- | --- | --- | --- | --- | --- |
|  | **Treatment** | | | **Pooled SEM** | ***P*-value** |
| Region of Interest | **CON** | **3ˊ-SL** | **6ˊ-SL** |  |  |
| *n* | 11 | 11 | 11 | **-** | **-** |
| Cerebellum | 0.257^a^ | 0.255^a^ | 0.238^b^ | 0.014 | **0.032** |
| Corpus callosum | 0.301 | 0.288 | 0.291 | 0.015 | 0.582 |
| Left caudate | 0.364 | 0.370 | 0.365 | 0.015 | 0.920 |
| Left hippocampus | 0.344^a^ | 0.332^ab^ | 0.316^b^ | 0.017 | **0.010** |
| Left internal capsule | 0.343 | 0.342 | 0.329 | 0.017 | 0.388 |
| Left cortex | 0.360 | 0.364 | 0.352 | 0.014 | 0.600 |
| Right caudate | 0.346 | 0.340 | 0.342 | 0.012 | 0.936 |
| Right hippocampus | 0.317 | 0.301 | 0.298 | 0.016 | 0.189 |
| Right internal capsule | 0.324 | 0.315 | 0.314 | 0.014 | 0.684 |
| Right cortex | 0.343 | 0.335 | 0.336 | 0.011 | 0.756 |
| Thalamus | 0.312 | 0.314 | 0.305 | 0.015 | 0.676 |
| White matter | 0.325 | 0.321 | 0.312 | 0.013 | 0.456 |
| Average AD mask | 0.293 | 0.288 | 0.281 | 0.010 | 0.350 |
| ^1^Data presented are least squares means and *P*-values from mixed model 1-way ANOVA. Abbreviations: PND, postnatal day; CON; control; 3ˊ-SL, 3ˊ-sialyllactose; 6ˊ-SL, 6ˊ-sialyllactose; SEM, standard error of mean. | | | | | |

| Supplemental Table 10. Radial diffusivity values (RD; x 10⁻³ /mm²/s) of pigs at PND 58^1^ | | | | | |
| --- | --- | --- | --- | --- | --- |
|  | **Treatment** | | | **Pooled SEM** | ***P*-value** |
| Region of Interest | **CON** | **3ˊ-SL** | **6ˊ-SL** |  |  |
| *n* | 11 | 11 | 11 | **-** | **-** |
| Cerebellum | 0.161 | 0.159 | 0.149 | 0.009 | 0.063 |
| Corpus callosum | 0.183 | 0.183 | 0.174 | 0.009 | 0.488 |
| Left caudate | 0.217 | 0.221 | 0.223 | 0.009 | 0.805 |
| Left hippocampus | 0.220^a^ | 0.213^ab^ | 0.204^b^ | 0.011 | **0.026** |
| Left internal capsule | 0.159 | 0.159 | 0.159 | 0.008 | 0.998 |
| Left cortex | 0.233 | 0.236 | 0.227 | 0.009 | 0.530 |
| Right caudate | 0.208 | 0.209 | 0.207 | 0.007 | 0.985 |
| Right hippocampus | 0.222 | 0.217 | 0.217 | 0.007 | 0.760 |
| Right internal capsule | 0.202 | 0.195 | 0.194 | 0.010 | 0.418 |
| Right cortex | 0.150 | 0.146 | 0.150 | 0.007 | 0.743 |
| Thalamus | 0.188 | 0.192 | 0.189 | 0.009 | 0.850 |
| White matter | 0.190 | 0.188 | 0.185 | 0.008 | 0.674 |
| Average RD mask | 0.181 | 0.178 | 0.174 | 0.006 | 0.393 |
| ^1^Data presented are least squares means and *P*-values from mixed model 1-way ANOVA. Abbreviations: PND, postnatal day; CON; control; 3ˊ-SL, 3ˊ-sialyllactose; 6ˊ-SL, 6ˊ-sialyllactose; SEM, standard error of mean. | | | | | |

| Supplemental Table 11. Mean diffusivity values (MD; x 10⁻³ /mm²/s) of pigs at PND 58^1^ | | | | | |
| --- | --- | --- | --- | --- | --- |
|  | **Treatment** | | | **Pooled SEM** | ***P*-value** |
| Region of Interest | **CON** | **3ˊ-SL** | **6ˊ-SL** |  |  |
| *n* | 11 | 11 | 11 | **-** | **-** |
| Cerebellum | 0.193^a^ | 0.191^a^ | 0.179^b^ | 0.011 | **0.048** |
| Corpus callosum | 0.219 | 0.213 | 0.212 | 0.011 | 0.679 |
| Left caudate | 0.266 | 0.271 | 0.271 | 0.011 | 0.896 |
| Left hippocampus | 0.261^a^ | 0.253^ab^ | 0.241^b^ | 0.013 | **0.017** |
| Left internal capsule | 0.220 | 0.220 | 0.215 | 0.011 | 0.765 |
| Left cortex | 0.275 | 0.279 | 0.269 | 0.011 | 0.560 |
| Right caudate | 0.254 | 0.253 | 0.252 | 0.009 | 0.985 |
| Right hippocampus | 0.262 | 0.257 | 0.257 | 0.008 | 0.758 |
| Right internal capsule | 0.240 | 0.230 | 0.229 | 0.012 | 0.297 |
| Right cortex | 0.208 | 0.202 | 0.205 | 0.009 | 0.775 |
| Thalamus | 0.229 | 0.233 | 0.228 | 0.011 | 0.831 |
| White matter | 0.235 | 0.233 | 0.227 | 0.010 | 0.571 |
| Average MD mask | 0.218 | 0.215 | 0.210 | 0.008 | 0.373 |
| ^1^Data presented are least squares means and *P*-values from mixed model 1-way ANOVA. Abbreviations: PND, postnatal day; CON; control; 3ˊ-SL, 3ˊ-sialyllactose; 6ˊ-SL, 6ˊ-sialyllactose; SEM, standard error of mean. | | | | | |

| Supplemental Table 12. Fractional anisotropy values (FA; arbitrary units) of pigs at PND 58^1^ | | | | | |
| --- | --- | --- | --- | --- | --- |
|  | **Treatment** | | | **Pooled SEM** | ***P*-value** |
| Region of Interest | **CON** | **3ˊ-SL** | **6ˊ-SL** |  |  |
| *n* | 11 | 11 | 11 | **-** | **-** |
| Cerebellum | 0.338 | 0.340 | 0.347 | 0.005 | 0.108 |
| Corpus callosum | 0.361 | 0.352 | 0.366 | 0.005 | 0.108 |
| Left caudate | 0.345 | 0.342 | 0.332 | 0.005 | 0.091 |
| Left hippocampus | 0.312 | 0.304 | 0.304 | 0.004 | 0.051 |
| Left internal capsule | 0.494 | 0.493 | 0.484 | 0.006 | 0.177 |
| Left cortex | 0.314 | 0.311 | 0.312 | 0.002 | 0.599 |
| Right caudate | 0.343 | 0.334 | 0.338 | 0.005 | 0.450 |
| Right hippocampus | 0.314^a^ | 0.301^b^ | 0.303^b^ | 0.004 | **0.033** |
| Right internal capsule | 0.492 | 0.496 | 0.484 | 0.007 | 0.117 |
| Right cortex | 0.311 | 0.312 | 0.313 | 0.002 | 0.733 |
| Thalamus | 0.354^a^ | 0.343^ab^ | 0.334^b^ | 0.004 | **0.011** |
| White matter | 0.373 | 0.373 | 0.371 | 0.004 | 0.727 |
| Average FA mask | 0.353 | 0.352 | 0.357 | 0.003 | 0.350 |
| ^1^Data presented are least squares means and *P*-values from mixed model 1-way ANOVA. Abbreviations: PND, postnatal day; CON; control; 3ˊ-SL, 3ˊ-sialyllactose; 6ˊ-SL, 6ˊ-sialyllactose; SEM, standard error of mean. | | | | | |

| **Supplemental Table 13.** Myelin water fraction of pigs at PND 58^1^ | | | | | |
| --- | --- | --- | --- | --- | --- |
|  | **Treatment** | | | **Pooled SEM** | ***P*-value** |
| **Region of Interest** | **CON** | **3ˊ-SL** | **6ˊ-SL** |  |  |
| *n* | 10 | 12 | 11 | - | - |
| Whole brain | 0.059 | 0.061 | 0.060 | 0.001 | 0.139 |
| Combined cortex | 0.056 | 0.058 | 0.057 | 0.010 | 0.072 |
| Combined hippocampus | 0.059 | 0.057 | 0.061 | 0.003 | 0.465 |
| Combined internal capsule | 0.098 | 0.103 | 0.101 | 0.002 | 0.298 |
| Cerebellum | 0.059 | 0.060 | 0.057 | 0.001 | 0.370 |
| Corpus callosum | 0.041 | 0.040 | 0.048 | 0.003 | 0.205 |
| Hypothalamus | 0.049 | 0.054 | 0.059 | 0.003 | 0.116 |
| Left caudate | 0.074 | 0.075 | 0.075 | 0.004 | 0.992 |
| Left cortex | 0.053 | 0.054 | 0.053 | 0.010 | 0.567 |
| Left hippocampus | 0.055 | 0.053 | 0.055 | 0.002 | 0.693 |
| Left inferior colliculi | 0.051 | 0.057 | 0.058 | 0.004 | 0.397 |
| Left internal capsule | 0.093 | 0.099 | 0.099 | 0.003 | 0.228 |
| Left olfactory bulb | 0.053 | 0.054 | 0.052 | 0.003 | 0.929 |
| Left putamen-globus pallidus | 0.085 | 0.090 | 0.091 | 0.003 | 0.375 |
| Left superior colliculi | 0.069 | 0.069 | 0.066 | 0.005 | 0.811 |
| Medulla | 0.062 | 0.062 | 0.065 | 0.002 | 0.430 |
| Midbrain | 0.066 | 0.068 | 0.071 | 0.002 | 0.083 |
| Pons | 0.066 | 0.067 | 0.067 | 0.002 | 0.686 |
| Right caudate | 0.077 | 0.078 | 0.086 | 0.004 | 0.243 |
| Right cortex | 0.057 | 0.061 | 0.061 | 0.001 | 0.064 |
| Right hippocampus | 0.062 | 0.060 | 0.064 | 0.003 | 0.609 |
| Right inferior colliculi | 0.060 | 0.062 | 0.057 | 0.004 | 0.699 |
| Right internal capsule | 0.101 | 0.106 | 0.104 | 0.003 | 0.527 |
| Right olfactory bulb | 0.055 | 0.054 | 0.058 | 0.003 | 0.474 |
| Right putamen-globus pallidus | 0.093^ab^ | 0.102^a^ | 0.090^b^ | 0.003 | **0.030** |
| Right superior colliculi | 0.067 | 0.073 | 0.078 | 0.003 | 0.051 |
| Thalamus | 0.088 | 0.091 | 0.093 | 0.002 | 0.118 |
| ^1^Data presented are least squares means and *P*-values from mixed model 1-way ANOVA.  Abbreviations: PND, postnatal day; CON; control; 3ˊ-SL, 3ˊ-sialyllactose; 6ˊ-SL, 6ˊ-sialyllactose; SEM, standard error of mean.  ^ab^Means lacking a common superscript letter within a row differ (*P* < 0.05). | | | | | |
